# Heart failure and preserved ejection fraction: pathophysiology, clinical assessment, and management of exercise intolerance

**DOI:** 10.1093/eurheartj/ehag175

**Published:** 2026-04-15

**Authors:** Isabela Landsteiner, Jan Verwerft, Dmitri Belov, Frederik H Verbrugge, Gregory D Lewis

**Affiliations:** Mass General Brigham Heart and Vascular Institute, Boston, MA 02114, USA; Department of Cardiology and Jessa & Science, Jessa Hospital, Hasselt, Belgium; Faculty of Medicine and Life Sciences/LCRC, UHasselt, Diepenbeek, Belgium; Mass General Brigham Heart and Vascular Institute, Boston, MA 02114, USA; Faculty of Medicine and Pharmacy, Vrije Universiteit Brussel, Brussels, Belgium; Centre for Cardiovascular Diseases, University Hospital Brussels, Jette, Belgium; Mass General Brigham Heart and Vascular Institute, Boston, MA 02114, USA

**Keywords:** Exercise test, Heart failure, preserved ejection fraction, Diastolic heart failure, Disease management, Therapeutics

## Abstract

Exercise intolerance is a clinical hallmark of heart failure with preserved ejection fraction (HFpEF) that confers high morbidity and predicts mortality. The mechanisms underlying exercise intolerance in HFpEF are diverse and often include compound deficits in multi-organ reserve capacity that culminate in marked functional limitations. This review describes aetiologies of exercise intolerance in HFpEF, tools to quantify relative physiologic deficits unmasked during exercise, and insights gained from interventional trials that have aimed to augment exercise capacity in HFpEF. The domain-based phenotyping approach described highlights the value of comprehensive phenotyping of both cardiac and extra-cardiac reserve capacity to advance understanding of how to deploy individualized interventions to bolster exercise tolerance in HFpEF.

## Graphical Abstract

**Graphical Abstract:**
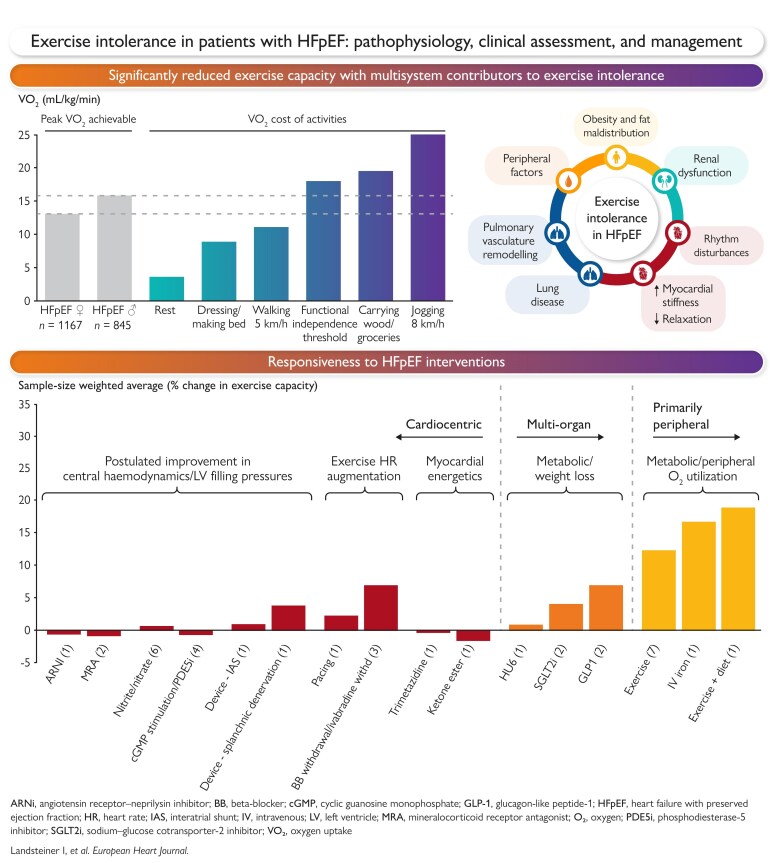
Multisystem mechanisms underlying exercise intolerance (EI) in heart failure with preserved ejection fraction (HFpEF) and differential responsiveness to therapeutic interventions. EI in HFpEF reflects multisystem impairments, resulting in markedly reduced peak VO₂ relative to the metabolic cost of common physical activities. Sample-size-weighted trial data show limited gains with primarily cardiocentric therapies, whereas exercise and metabolic/peripheral-targeted interventions tend to yield greater improvements in functional capacity. Numbers in parentheses indicate the number of trials included for each intervention category.

## Introduction

Heart failure (HF) is a major and growing global public health challenge, currently affecting over 64 million individuals worldwide.^1^ HF with preserved ejection fraction (HFpEF) accounts for nearly half of all HF cases and continues to increase in prevalence.^1,2^ Clinical characteristics and risk factor profiles of HFpEF have considerable regional heterogeneity, shaped by demographic trends, comorbidity patterns, and environmental exposures.^1,3,4^

**Figure 1:**
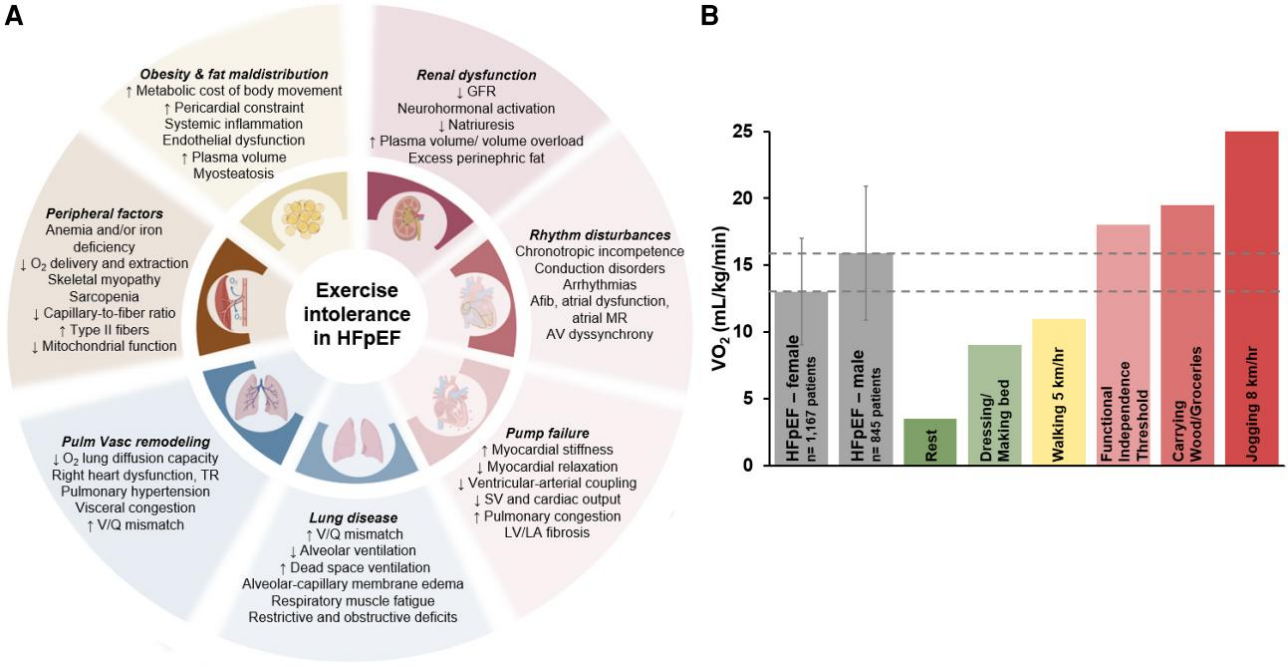
Exercise Intolerance in HFpEF. (*A*) Schematic representation of organ system impairments contributing to exercise intolerance in HFpEF. Each domain reflects distinct pathophysiologic mechanisms limiting oxygen delivery and/or utilization during exertion. (*B*) Mean peak VO_2_ (ml/kg/min) in HFpEF patients by sex, compared with the metabolic demands of daily activities. Values represent mean and standard deviation aggregated from eight studies reporting sex-stratified peak VO_2_ in HFpEF. Abbreviations: Afib, atrial fibrillation; AV, atrioventricular; CO, cardiac output; GFR, glomerular filtration rate; LA, left atrium; LV, left ventricle; MR, mitral regurgitation; O_2_, oxygen; PV, pulmonary-vascular; SV, stroke volume; TR, tricuspid regurgitation; V/Q, ventilation/perfusion; VO_2_, oxygen uptake

Despite regional variation in clinical presentation, exercise intolerance (EI) is consistently observed in HFpEF, even in the absence of overt evidence of congestion at rest.^5^ EI reflects impairments in both central and peripheral reserve capacity, with most patients exhibiting compound deficits across cardiovascular, pulmonary, haematologic, and neuromuscular systems (*Figure 1A*).^6,7^ The various admixtures of these deficits underscore the systemic and heterogeneous nature of the syndrome.^6,8,9^ This evolving understanding of HFpEF as a disorder of multisystem function—with exercise limitation as arguably its most sensitive and primary manifestation—provides a strong rationale for pursuing exercise-based physiological assessments that unmask and rank-order impairments that may not be evident at rest.^10–12^ Exercise-based phenotyping thereby offers critical insights into diagnosis and risk stratification of HFpEF, while also potentially informing targeted treatments.^4,13–17^

## Extent and aetiology of exercise intolerance in HFpEF

Peak oxygen uptake (pVO_2_) represents a gold standard measurement of functional capacity in HFpEF and, when interpreted in the context of the metabolic demands of daily activities, underscores the marked exercise limitation faced by this population. Aggregated data from eight studies reporting sex-specific pVO_2_ values in 2012 HFpEF patients show that women with HFpEF achieve a mean (±standard deviation) pVO_2_ of approximately 13.1 ± 4.1 mL/kg/min, while men achieve a pVO_2_ of 15.9 ± 5.5 mL/kg/min (*Figure 1B*).^18–26^ Common daily activities, such as walking at 5 km per hour (km/hr), impose a metabolic cost equivalent of >70% of the average pVO_2_ that patients living with HFpEF can afford. More vigorous tasks, like jogging at 8 km/hr, exceed the achievable metabolic capacity in most HFpEF patients.^27^ Notably, pVO_2_ values in HFpEF are comparable to thresholds used to trigger evaluation of patients with HF with reduced ejection fraction (HFrEF) for advanced therapies such as heart transplantation, underscoring the severity of exercise limitation in HFpEF.^28,29^

EI in HFpEF is distinct from well-demarcated conditions such as acute coronary syndromes, clearly delineated by biomarker elevations, that impose limitations on exercise capacity attributable to reduced cardiac performance. In HFpEF, causes of EI are shaped by less distinct, cumulative lifetime exposures such as limited physical activity, systemic inflammation, and excess or maldistributed adiposity that collectively contribute to impaired cardiac performance while also independently conferring functional limitations (*Figure 1A*).^6,8^ HFpEF has been characterized as an ‘exercise deficiency syndrome’ in which lack of exercise predisposes to small ventricular chamber size, cardiac atrophy, and increased stiffness.^30,31^ Population studies have found that cardio-specific abnormalities, such as left bundle branch block or infarct patterns on electrocardiogram, heighten hazard ratio for future development of HFrEF > HFpEF, whereas midlife adiposity and sedentary lifestyle predispose to HFpEF > HFrEF.^30^ As a result, assessment of broad metabolic and multi-organ function is requisite to understanding EI in HFpEF.

### Cardiac contributions

In HFpEF, impaired myocardial relaxation (prolonged Tau) and heightened stiffness (steeper slope of the end-diastolic pressure volume relationship) predispose to elevated filling pressures during exercise. Normal individuals demonstrate improved LV relaxation with exercise, whereas in HFpEF, Tau fails to shorten normally with exercise despite physiologic demand for enhanced relaxation as heart rate ascends.^32,33^ Additional cardiac abnormalities (summarized in *Figure 1A*) include pericardial constraint, diminished contractile reserve, and chronotropic incompetence coupled with impaired atrial capacitor and booster function that lead to exaggerated ascent in cardiac filling pressures during exercise.^34^

**Figure 2:**
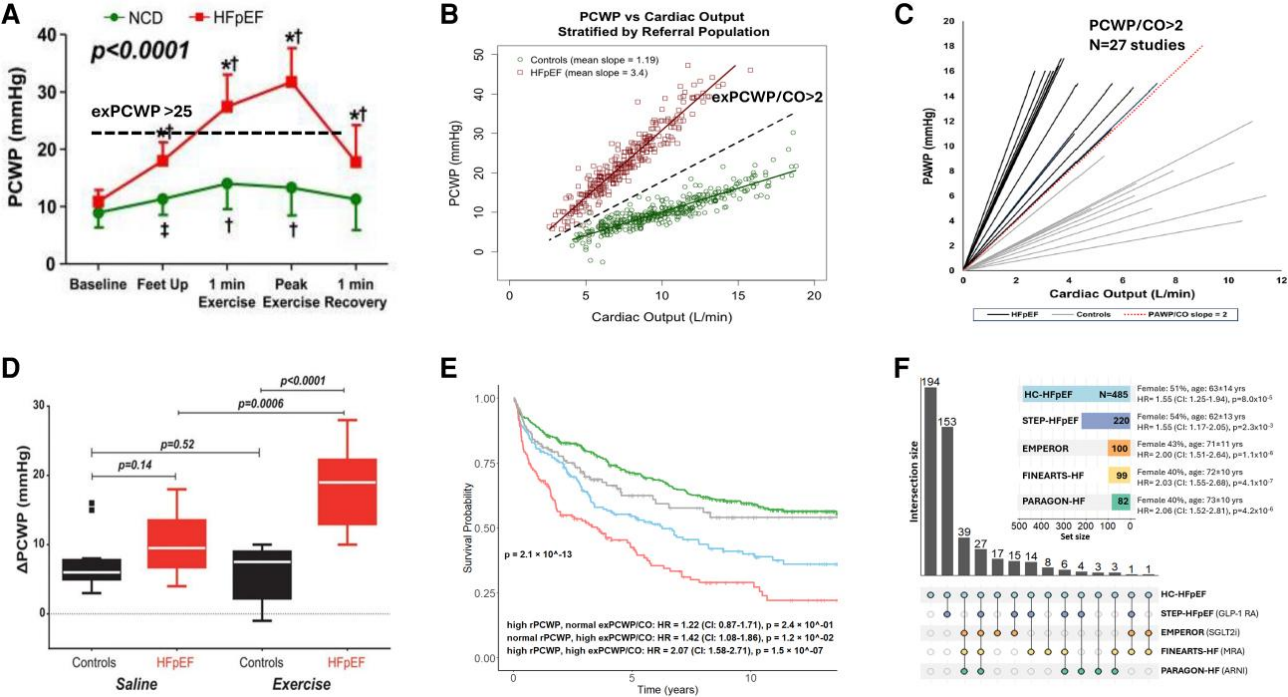
Prognostic implications of resting and exercise haemodynamic profiles in HFpEF. (*A*) Pulmonary capillary wedge pressure (PCWP) rises more markedly in HFpEF (red) than in non-cardiac dyspnoea (NCD, green) during leg elevation and throughout exercise. *Adapted from Borlaug et al with permission.*^35^ (*B*) Relationship between PCWP and CO in individuals with HFpEF and healthy controls. The dashed line indicates a PCWP/CO slope of 2 mmHg/L/min, which effectively separates the two groups. *Adapted from Einsman et al. with permission.*^11^ (*C*) PCWP to CO slopes in cohorts of patients with HFpEF and healthy controls. The red line denotes the upper limit of normal for the PCWP/CO slope (>2 mmHg/L/min). *Adapted from Baratto et al with permission.*^36^ (*D*) Tukey boxplot illustrating changes in PCWP with exercise and saline infusion. Exercise-induced increases in PCWP were significantly greater in HFpEF (red) compared with controls (black), while PCWP responses to saline were similar between groups. *Adapted from Andersen et al. with permission.*^37^ (*E*) Kaplan–Meier curve and Cox Proportional Hazard Model for all-cause mortality and cardiovascular events comparing haemodynamic profiles to the group with normal rPCWP (supine resting PCWP <15 mmHg) and normal exPCWP/CO (PCWP/CO slope ≤2 mmHg/L/min), adjusted for age, sex, body mass index (BMI), and NT-proBNP (N-terminal pro-B-type natriuretic peptide). *Adapted from Landsteiner et al with permission.*^10^ (*F*) Among individuals with haemodynamically confirmed HFpEF (HC-HFpEF), the UpSet plot displays overlap in eligibility across different clinical trial criteria. Horizontal bars show the total number of patients meeting individual trial entry criteria, while vertical bars depict the number meeting specific combinations of criteria. For each subgroup, the proportion of female participants, mean age ± standard deviation, and hazard ratios (HRs) for a composite outcome of all-cause mortality and cardiovascular events are provided. HRs were calculated using Cox proportional hazards models adjusted for age, sex, BMI, and NT-proBNP, comparing those who met vs did not meet each criterion within the cohort. *Adapted from Landsteiner et al with permission.*^10^ Abbreviations: CI, confidence interval; CO, cardiac output; rPCWP, resting pulmonary capillary wedge pressure; exPCWP, exercise pulmonary capillary wedge pressure; GLP-1 RA, glucagon-like peptide-1 receptor agonist; HC-HFpEF, haemodynamically confirmed HFpEF; HFpEF, heart failure with preserved ejection fraction; HR, hazard ratio; MRA, mineralocorticoid receptor antagonist; NCD, non-cardiac dyspnoea; SGLT2i, sodium-glucose cotransporter-2 inhibitor

Pulmonary capillary wedge pressure (PCWP) ≥ 25 mmHg during supine exercise serves as a diagnostic criterion for HFpEF (*Figure 2A*).^35^ In addition to absolute elevations in filling pressure, HFpEF is characterized by impaired augmentation of cardiac output (CO) during exercise (*Figure 2*).^38^ This can be expressed as a steep PCWP/CO relationship, with PCWP/CO slope ≥ 2 mmHg/L/min constituting an abnormal threshold (*Figure 2A*–*F*)^11,36^ and (Supplementary data online, *Table S1*).

Building on invasive pressure–flow indices, Tan *et al*. showed that exercise limitation in HFpEF reflects combined systolic and diastolic abnormalities, involving impaired myocardial deformation and twist–untwist mechanics that lead to reduced ventricular suction, delayed untwisting, and impaired early diastolic filling.^39^ Patients with HFpEF exhibited reduced longitudinal and radial strain, diminished apical rotation, and blunted systolic functional reserve, with reduced and delayed untwisting, impaired LV suction, and higher filling pressures during exercise, supporting HFpEF as not an isolated disorder of diastole.^39^

### Systemic vascular contributors

Arterial stiffness is a key systemic contributor to EI in HFpEF. It arises from the interplay of vascular ageing, neurohormonal activation, inflammation, and structural remodelling of the arterial wall.^40^ Arterial stiffness reduces aortic compliance, increases left ventricular (LV) afterload, and increases late systolic wave reflection, thereby impairing diastolic relaxation and contractile efficiency. Indices of ventricular–arterial (V–A) coupling integrate these interactions, and elevated vascular stiffness has consistently been associated with impaired LV reserve.^41,42^ Impairments in endothelial function and microvascular reactivity further limit skeletal muscle and coronary perfusion, correlating with greater symptom burden and reduced functional capacity.^43,44^ Consistent with these observations, direct measurements during exercise have demonstrated that patients with HFpEF may develop transient myocardial injury related to coronary supply–demand mismatch, which is associated with reduced peak VO_2_ and limitations in cardiac reserve.^45^

Derived indices of vascular function provide further prognostic insight in HFpEF. Reddy *et al*. demonstrated that during submaximal exercise, patients with HFpEF exhibit lower total arterial compliance and higher effective arterial elastance compared with controls, despite similar mean arterial pressures.^46^ These abnormalities were directly associated with higher ventricular filling pressures and reduced cardiac output.^46^ Complementing these findings, Namasivayam *et al*. reported that greater inducible blood pressure pulsatility in HFpEF reflects increased arterial stiffness and is independently associated with a higher risk of adverse cardiovascular outcomes.^42^

### Pulmonary and pulmonary vascular contributions

Among patients with HFpEF, 94% manifest abnormalities in one or more pulmonary function tests, and intrinsic properties of the pulmonary vasculature, such as pulmonary distensibility, are often impaired.^47,48^ Pulmonary vascular distensibility, defined as the per cent increase in pulmonary vessel diameter per mm Hg increase in pressure, is closely linked to impaired RV contractile reserve during exercise and is reduced in HFpEF.^48^ Abnormal PAP-flow relationships with exercise-induced right ventricular–pulmonary artery (RV–PA) uncoupling have also been described in HFpEF.^49^ Together, these abnormalities link pulmonary vascular dysfunction to impaired cardiac output reserve and reduced aerobic capacity during exertion.

HFpEF patients frequently experience impaired ventilatory efficiency as indicated by increased minute ventilation/carbon dioxide production (VE/VCO_2_) slope (Supplementary data online, *Table S1*), reflecting abnormalities in both component variables (fractional dead space and partial pressure of carbon dioxide [PaCO_2_] set point, *Figure 1A*). When coupled with frequent obstructive deficits in HFpEF that limit forced expiratory volume,^50^ HFpEF patients can reach their pulmonary mechanical limit during exercise, as denoted by minute ventilation approaching maximum voluntary ventilation during incremental exercise (Supplementary data online, *Table S1*). Rapid shallow breathing in the context of reduced respiratory muscle strength further contributes to increased physiologic dead space and inefficient ventilation in HFpEF.

Impaired pulmonary gas exchange with reduced diffusing capacity for carbon monoxide (DLco) has been observed at rest and during exercise in HFpEF. This impairment is primarily attributable to reductions in alveolar-capillary membrane conductance (Dm), with a variable contribution from pulmonary capillary blood volume (Vc).^51–53^ Chronic elevation of left-sided filling pressures in HFpEF leads to pulmonary vascular remodelling, increased extravascular lung water, and thickening of the alveolar-capillary membrane, all of which contribute to impaired gas transfer.^52,53^ During exercise, the inability to appropriately augment Dm and Vc further limits oxygen uptake and contributes to exertional dyspnoea. Notably, the reduction in DLco in HFpEF may be out of proportion to the degree of pulmonary hypertension or left atrial pressure elevation, implicating intrinsic alveolar-capillary dysfunction.

**Figure 3:**
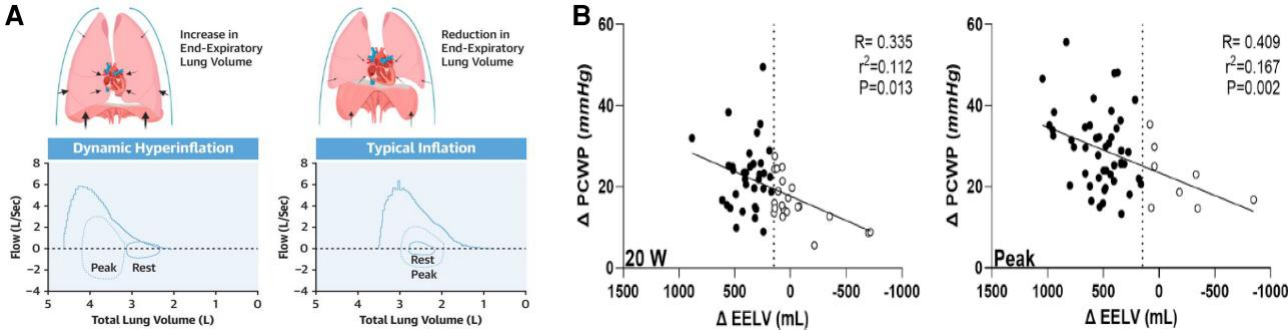
Dynamic Hyperinflation and Correlation to Exercise PCWP in HFpEF. (*A*) Illustration and representative flow-volume loops from a patient exhibiting dynamic hyperinflation (left) and typical inflation (right). The thick outermost line indicates the maximal expiratory flow. Smaller loops depict resting tidal breathing, while the larger dashed loops correspond to flow–volume loops at peak exercise. *Adapted from Leahy et al with permission.*^54^ (*B*) Relationship of the difference of PCWP from rest to 20-W and peak exercise. Vertical dotted lines represent the qualifying threshold for dynamic hyperinflation (an increase in EELV ≥150 mL from rest), with individuals who dynamically hyperinflate (solid circles) and with typical inflation (open circles). Solid lines represent linear regressions of the sample (*n* = 55). *Adapted from Leahy et al with permission.*^54^

Importantly, extracardiac thoracic mechanics may influence the interpretation of left-sided filling pressures during exercise. In the study by Leahy *et al*., dynamic hyperinflation quantified by inspiratory-capacity manoeuvres and exercise flow–volume loop analysis was associated with higher PCWP at a standardized submaximal workload and at peak exercise (*Figure 3*).^54^ Both increased end-expiratory lung volume and elevated PCWP may also reflect shared associations with greater adiposity.^55^ Consistent with these novel findings, Campain *et al*. reported that, at peak exercise, lower resting per cent predicted FEV1 was independently related to the greater degree of respirophasic variation in PCWP tracings.^56^

### Peripheral-skeletal contributors

#### Skeletal muscle oxygen extraction and utilization

**Figure 4:**
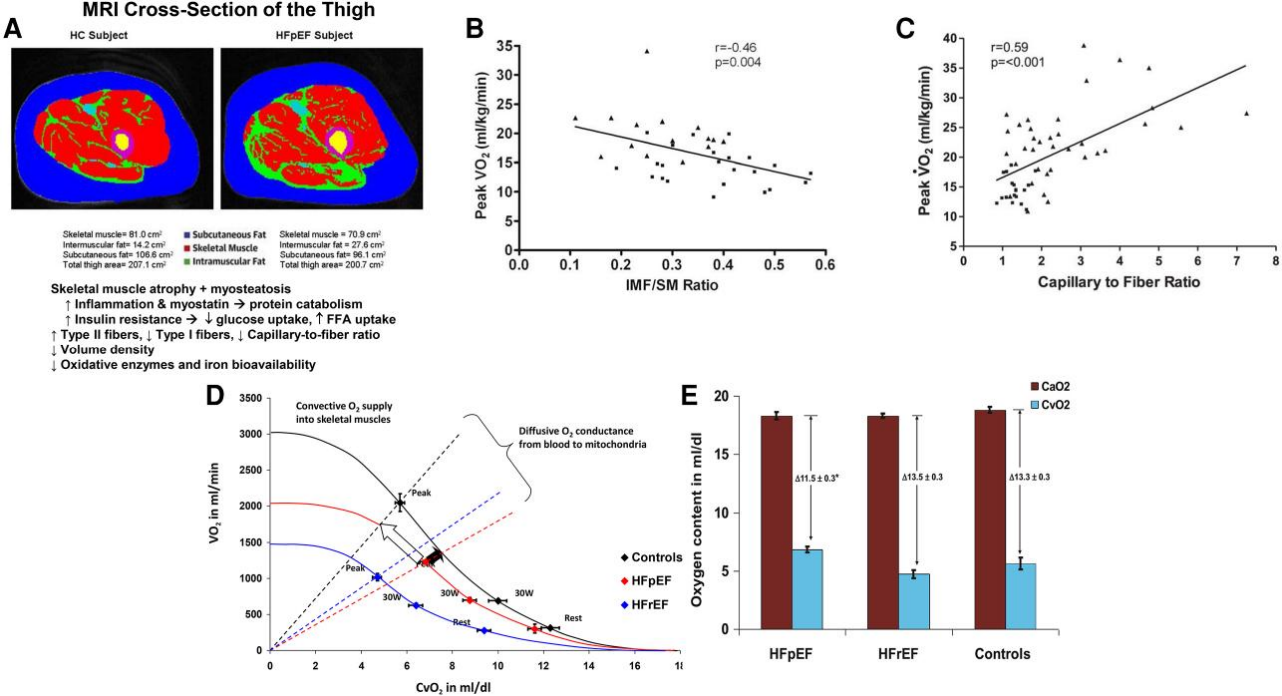
Peripheral contributors to exercise intolerance in HFpEF. (*A*) Axial magnetic resonance imaging of the mid-thigh in a patient with HFpEF and a healthy control. Skeletal muscle is shown in red, intermuscular fat in green, subcutaneous fat in blue, femoral cortex in purple, and femoral medulla in yellow. The HFpEF subject demonstrates increased intermuscular fat compared with the healthy control, despite similar subcutaneous fat. *Adapted from Haykowsky et al. with permission.*^57^ (*B*) Plot representing the relationship between intermuscular fat/skeletal muscle ratio and peak VO_2_ in HFpEF (solid squares) and healthy controls (solid triangles). *Adapted from Haykowsky with permission.*^57^ (*C*) Plot representing the relationship between capillary to fibre ratio and peak VO_2_ in HFpEF (solid squares) and healthy controls (solid triangles). *Adapted from Haykowsky et al with permission.*^57^ (*D*) This schematic illustrates the convective and diffusive components that interact to determine oxygen uptake (VO_2_) during exercise in patients with HFpEF, HFrEF, and controls. Mean values for mixed venous oxygen content (CvO_2_) and VO_2_ at rest, 30 W, and peak exercise are used to construct Fick principle lines, which reflect convective oxygen delivery and are curvilinear because they reflect the haemoglobin dissociation curve. Vertical lines extending from the origin to the VO_2_–CvO_2_ plot at peak exercise represent maximum diffusive oxygen delivery, with a steeper relationship indicating better oxygen diffusion. The black arrow illustrates the potential improvement in peak VO_2_ in HFpEF if convective oxygen delivery was normalized to that of controls; the white arrow shows the improvement in peak VO_2_ if diffusive conductance was normalized. *Adapted from Dhakal et al with permission.*^58^ (*E*) Arterial oxygen content (CaO_2_) and mixed venous oxygen content (CvO_2_) at peak exercise in HFpEF, HFrEF, and controls. *P-value <.05 for comparison of HFpEF with HFrEF and controls. *Adapted from Dhakal et al with permission.*^58^ Abbreviations: CaO_2_, arterial oxygen content; CvO_2_, venous oxygen content; FFA, free fatty acids; HC, healthy control; HFpEF, heart failure with preserved ejection fraction; HFrEF, heart failure with reduced ejection fraction; IMF, intermuscular fat; MRI, magnetic resonance imaging; myosteatosis, intramuscular fat infiltration; O_2_, oxygen; SM, skeletal muscle; VO_2_, oxygen consumption

Peripheral abnormalities, particularly within skeletal muscle, have been extensively investigated and significantly contribute to EI in HFpEF, including myosteatosis, sarcopenia, capillary rarefaction, increased vascular stiffness, reduced type I (oxidative) muscle fibres, and reduced type I-to-type II fibre ratio (*Figures 1A* and *4A*).^59,60^ The extent of these peripheral abnormalities has been shown to directly correlate with the extent of reduction in pVO_2_ (*Figure 4B* and *C*).^61^

While direct measurement of arteriovenous oxygen content difference (Ca–vO_2_) during invasive cardiopulmonary exercise testing (CPET) permits assessment of the contribution of peripheral O_2_ extraction to pVO_2_, it is important to account for the influence of rate of O_2_ delivery on Ca-vO_2_. Skeletal muscle oxygen diffusion capacity (DmO_2_), approximated by the VO_2_/mixed venous oxygen tension (PvO_2_) ratio, specifically represents the peripheral oxygen utilization. For example, a patient with HFpEF with low CO reserve may exhibit a high Ca–vO_2_ simply due to prolonged capillary transit time, masking a true DmO_2_ deficit (*Figure 4D* and *E*).^6^

Anaemia and iron deficiency, which are present in >50% of patients with HFpEF, can also contribute to impaired oxygen delivery and reduced peripheral oxygen utilization.^62^ Iron plays a central role not only in haemoglobin-mediated oxygen transport but also as a cofactor in mitochondrial respiration, oxidative phosphorylation, and key enzymatic reactions of the citric acid cycle, all of which are essential to support efficient tissue oxygen metabolism.^63^ Iron deficiency, particularly when defined by transferrin saturation <20%, is associated with reduced pVO_2_ and worse clinical outcomes in HFpEF.^64–66^

#### Obesity

Obesity in HFpEF is often marked by excess visceral and epicardial fat, which promotes systemic inflammation, myocardial remodelling, microvascular dysfunction, and increased pericardial restraint (*Figure 1A*).^55^ These alterations contribute to impaired diastolic reserve and elevated cardiac filling pressures during exercise.^67^ Pulmonary mechanics are also compromised by excess thoracic and abdominal adiposity, which restricts chest wall expansion, increases the work of breathing, and reduces ventilatory reserve.^68,69^

**Figure 5:**
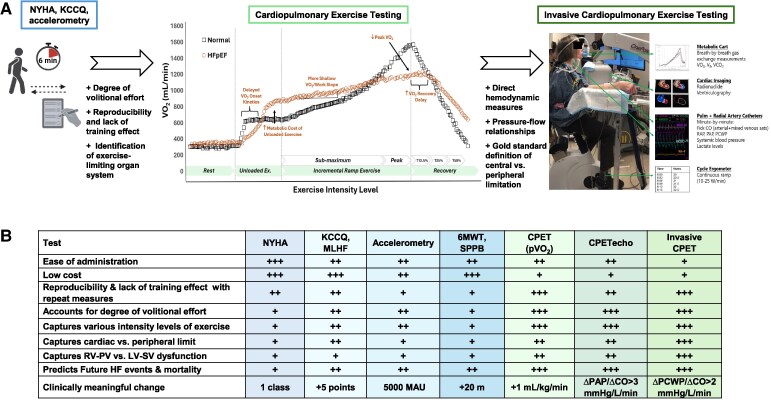
Evaluation of functional capacity and physiologic impairment in HFpEF. (*A*) Incremental values of different clinical tools used to assess exercise capacity. Middle figure showing VO_2_ trajectory measured by CPET across rest, unloaded exercise, incremental ramp exercise, and recovery in a representative patient with HFpEF (orange) and a healthy control (black). Key physiologic abnormalities in HFpEF include delayed VO_2_ kinetics, increased VO_2_ during unloaded activity, reduced VO_2_/work slope and peak VO_2_, and prolonged VO_2_ recovery. Right upper corner figure showing invasive CPET protocol at Massachusetts General Hospital. (*B*) Comparative overview of clinical tools used to assess functional capacity in HFpEF. Plus signs indicate relative strength across listed categories. Abbreviations: 6MWT, 6-minute walk test; CO, cardiac output; CPET, cardiopulmonary exercise testing; CPETecho, CPET with echocardiography; Ex, exercise; HF, heart failure; HFpEF, heart failure with preserved ejection fraction; KCCQ, Kansas City Cardiomyopathy Questionnaire; LV–SV, left ventricle–systemic vasculature; MAU, monitor activity units; m, metres; mph, miles per hour; MLHFQ, Minnesota Living with Heart Failure Questionnaire; NYHA, New York Heart Association; PAP, pulmonary artery pressure; PCWP, pulmonary capillary wedge pressure; pVO_2_, peak oxygen consumption; RV–PV, right ventricle–pulmonary vasculature; SPPB, Short Physical Performance Battery; VO_2_, oxygen consumption

In addition to central effects, obesity imposes significant peripheral limitations. Adipose infiltration into skeletal muscle, capillary rarefaction, and mitochondrial dysfunction impair muscle perfusion and oxygen extraction during activity (*Figure 4B* and *C*).^67^ Furthermore, obesity increases the metabolic cost of initiating movement—referred to as internal work— a body mass index (BMI)-related measure that quantifies the work equivalents required to initiate unloaded exercise (*Figure 5A*, **unloaded exercise**).^70^

## Clinical assessment in HFpEF

Dynamic, integrative assessments—such as cardiopulmonary exercise testing (CPET) and exercise stress echocardiography—aid in the early detection of abnormalities present during exercise in both central and peripheral organ systems in HFpEF (Supplementary data online, *Table S1* and *Figure 5*). These modalities have the potential to unmask haemodynamic and peripheral deficits that are not ascertainable with resting assessments but are relevant to EI.

### Global exercise capacity and symptom burden

Evaluation of EI in HFpEF should combine patient-reported outcomes and objective tests. The Kansas City Cardiomyopathy Questionnaire (KCCQ) offers reproducible insight into symptom burden and quality of life, and outperforms other patient-reported outcomes in HFpEF while also offering greater granularity than medical provider-determined New York Heart Association (NYHA) classification (*Figure 5B*).^71,72^

Objective assessments of EI in HFpEF include the 6-min walk test (6MWT), accelerometry measures of physical activity, and CPET. While 6MWT is low-cost and broadly available, it does not quantify peak aerobic capacity or delineate the organ system that limits exercise. CPET delivers detailed physiological profiling, including pVO_2_ and ventilatory response, with respiratory exchange ratio (RER) values in excess of 1.05, helping to verify maximal effort.^14^ Accelerometers offer real-world physical activity data, though they are limited by wear variability and contextual interpretation.

Clinically meaningful thresholds for KCCQ, pVO_2,_ and 6MWT are useful for interpreting therapeutic response.^73^ These thresholds are summarized in *Figure 5B*, which illustrates the minimal changes associated with clinical benefit. It is important to note that correlations between NYHA class, KCCQ values, and objective measures like 6MWT or peak VO_2_ are modest. The potent prognostic performance of measured exercise variables, coupled with the modest ability to extrapolate what objective exercise measures will be from patient/provider perceptions, highlights the importance of performing objective measures of EI in HFpEF.^14^

### Domain-based assessment

**Figure 6:**
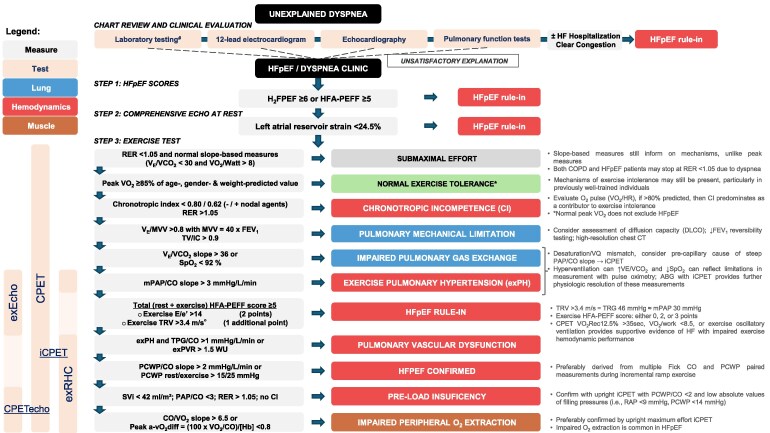
Clinical assessment flow-chart for domain-based diagnostic assessment in unexplained dyspnoea/HFpEF. Diagnostic measures uncover distinct mechanisms of exercise intolerance, grouped by physiological domain: haemodynamic (red), pulmonary (blue), and peripheral/muscular (brown). These mechanisms often co-exist, and tests are sequenced stepwise, beginning with resting evaluation (echocardiography, natriuretic peptides, HFpEF scores) and followed by dynamic testing in patients with unexplained exertional symptoms. Patients with known HFpEF may undergo the same structured assessment if symptoms persist despite therapy. CPET alone captures only pulmonary limitations and chronotropic incompetence (CI); exercise echocardiography or invasive testing is required to assess haemodynamic impairment. CPETecho and iCPET offer integrated, domain-spanning assessment. Submaximal testing may still yield diagnostic information via slope-based, effort-independent metrics. Key integrative markers include the PAP/CO slope (reflecting total pulmonary resistance, combining left atrial pressure and pulmonary vascular resistance) and the CO/VO_2_ ratio, a surrogate for peripheral oxygen extraction—both derivable from CPETecho or iCPET. ^#^Total blood count, C-reactive protein, iron, ferritin, transferrin saturation, HbA1c, serum creatinine, NT-proBNP. Abbreviations: PFT, pulmonary function testing; RHC, right heart catheterization; CPET, cardiopulmonary exercise testing; ExEcho, exercise echocardiography; CPETecho, combined cardiopulmonary exercise testing and echocardiography; iCPET, invasive cardiopulmonary exercise testing; PAP, pulmonary artery pressure; CO, cardiac output; VO_2_, oxygen uptake; VE, minute ventilation; VCO_2_, carbon dioxide output; VT, ventilatory threshold; a–vO_2_Diff, arteriovenous oxygen content difference; SpO_2_, peripheral oxygen saturation; MVV, maximal voluntary ventilation; FEV_1_, forced expiratory volume in 1 s; TV/IC, tidal volume to inspiratory capacity ratio; RER, respiratory exchange ratio; CI, chronotropic incompetence

Given the multisystem nature of HFpEF and the peril of assuming that an abnormal finding at rest explains EI, a domain-based approach to mapping haemodynamic, pulmonary, and peripheral abnormalities during exercise is warranted (*Figures 1A* and *6*). Such an approach can augment sensitivity to detect haemodynamically confirmed HFpEF in some patients while redirecting attention to extra-cardiac predominant deficits in other patients that can be prioritized for intervention.

#### Haemodynamic domain

##### Resting echocardiography

Resting echocardiography provides key insights into diastolic function, atrioventricular synchronicity, atrial function, and structural disease, and can help to identify HFpEF mimickers. Left atrial reservoir strain (LArS) < 24.5% enhances diagnostic accuracy for HFpEF (Supplementary data online, *Table S1* and *Figure 6*),^74^ as this threshold predicts elevated filling pressures not only at rest but also during exercise—unlike other studies that validated lower cutoffs (e.g. 18%) solely against resting haemodynamics.^75^

##### Natriuretic peptides

Natriuretic peptides such as N-terminal pro-B-type natriuretic peptide (NT-proBNP) help identify high-risk patients, but normal values do not exclude HFpEF. Many patients with haemodynamically confirmed HFpEF unmasked during exercise fall below NT-proBNP thresholds required by clinical trials (*Figure 2F*).^10^

##### Diagnostic HFpEF scores

Probability scores such as H_2_FPEF, HFpEF-ABA, and HFA-PEFF were derived and validated using the criterion of supine PCWP ≥ 25 mmHg measured at peak exercise.^76^ While useful in estimating the likelihood of HFpEF, this reference standard has potential limitations. Recent data derived from patients who performed both supine and upright exercise showed that up to half of patients who meet the supine threshold fail to meet criteria during upright exercise.^77^ These ‘discordant’ patients had fewer structural and haemodynamic abnormalities and lower H_2_FPEF scores compared with patients with concordant abnormal responses,^77^ suggesting that further investigation of both upright and supine exercise to refine HFpEF characterization is warranted.

##### Exercise echocardiography

###### Diastolic stress testing

Septal or average E/e’ ≥ 15 suggests impaired diastolic reserve (Supplementary data online, *Table S1*). However, the feasibility of E/e’ assessment declines with increasing workload, primarily due to E–A wave fusion, remaining high during low-level exercise (96.3% at 20 W) but decreasing at peak exercise (74.9%).^78^ In invasive–echocardiographic comparisons, E/e′ > 15 at 20 W showed a high positive predictive value for elevated filling pressures (PCWP ≥25 mmHg; ∼82%), whereas the negative predictive value of a ‘negative’ diastolic stress test (E/e′ ≤ 15) was limited (∼58%), with ∼42% of such patients still exhibiting PCWP ≥25 mmHg. Importantly, PCWP decreases rapidly during early recovery, whereas E/e’ frequently remains ≥15 in patients with abnormal exercise responses, indicating delayed normalization of diastolic relaxation rather than persistent elevation of filling pressures. Accordingly, recovery E/e’ should not be interpreted as a direct surrogate of contemporaneous wedge pressure, and E/e’ should be integrated with complementary exercise-derived parameters within a multiparametric framework.

Fusion of E and A waves at high heart rate (HR) (>90–100 bpm) may confound interpretation, highlighting the importance of ascertainment during submaximum exercise. Post-exercise imaging may also be considered given the prolonged elevation. Importantly, diastolic stress testing using E/e’ thresholds may fail to identify a subset of patients with invasively confirmed HFpEF, with prior studies demonstrating missed diagnoses in approximately one-quarter of cases, underscoring the need for cautious interpretation and, when diagnostic uncertainty persists, consideration of invasive exercise haemodynamic assessment.^78,79^ Stroke volume can be calculated from the LV outflow tract (LVOT) velocity–time integral (VTI) and diameter. The LVOT diameter, measured at rest, introduces the largest variability—especially since it is squared in the formula. Despite this, exercise-derived CO by echocardiography remains reliable, correlates well with cardiac magnetic resonance imaging (MRI),^80^ and is particularly relevant when changes in pressures relative to changes in CO are the focal point during exercise (Supplementary data online, *Table S1*).

###### Pulmonary hypertension during exercise (PAP/CO slope)

The pulmonary artery pressure (PAP)/CO slope quantifies total pulmonary resistance, integrating both left atrial pressure and pulmonary vascular resistance.^81,82^ A slope >3 mmHg/L/min indicates abnormal pressure–flow coupling during exercise (*Figure 6*), which does not delineate relative pre- and post-capillary contributions and should not be interpreted as a standalone non-invasive diagnostic criterion for HFpEF.^83^ Rather, it serves as an integrative barometer of abnormal exercise haemodynamic response. Both exercise echocardiography and invasive CPET (iCPET) assess this physiological parameter with generally good agreement in measuring pressure–flow relationships.^84^ iCPET is limited to specialized centres, while exercise echocardiography—especially using contrast-enhanced, semi-supine protocols—improves feasibility (up to 93%) and broadens access to this robust diagnostic and prognostic parameter for patients with exertional intolerance.^83,85,86^

Invasively, PAP/CO slope is most robust when derived from multiple datapoints across progressive stages of exercise, as this approach improves the precision of the slope estimate and minimizes the influence of measurement variability. Frequent sampling enables repeated measures prior to peak exercise, when the degree of respirophasic variation can be highly variable. Although slopes can be calculated from fewer measurements, the acquisition of a greater number of data points may enhance reproducibility and reduce the risk of misclassification.

Non-invasive estimation uses the Chemla formula from tricuspid regurgitation velocity (TRV) and cardiac output from LVOT diameter and VTI.^87^ TRV signal quality is enhanced with agitated colloid or saline and multipoint acquisition. Use of contrast should be routine, even when the resting signal appears adequate, as signal quality during exercise is often unpredictable. Measurements at intermediate workloads are valuable, as peak signals may be suboptimal; in such cases, a two-point slope (e.g. rest to intermediate stage) or PAP/CO ratio offers more diagnostic insight than isolated submaximal TRV readings. Including estimated right atrial pressure does not improve correlation with invasive PAP/CO slope or clinical outcomes.^80,88^ Multicentre data show that single-point non-invasive mPAP/CO ratios obtained during exercise provide prognostic performance comparable to multipoint slope-based approaches.^89^

Non-invasive mPAP/CO estimation can be used for screening and risk stratification, and to prompt confirmatory invasive evaluation.^81^ The mPAP/CO slope was estimated using the Chemla relation (mPAP = 0.61 × tricuspid regurgitation gradient [TRG] + 2 mmHg), applying the original formulation—derived from invasively measured systolic pulmonary artery pressure—to Doppler-derived TRG as a pragmatic adaptation for exercise echocardiography.^80,87^ TR signal quality was optimized with agitated colloid or saline, semi-supine position, and multi-point acquisition.^80,85,90^ Measurements were obtained at rest, submaximal, and peak; when peak TR was inadequate, intermediate-stage data were retained, and a two-point slope or single-point mPAP/CO ratio was calculated. Recent multicentre work shows that these simplified approaches provide prognostic information comparable to classical three-point regression.^89^

Chemla-derived mPAP has been validated during exercise, showing excellent correlation with simultaneous invasive measurements.^80,91^ Because resting right atrial pressure (RAP) does not reliably reflect RAP during exercise, incorporating a resting RAP estimate does not materially improve agreement between Doppler-derived and invasively measured PAP and prognostic discrimination.^80,88,92^ In HFpEF, atrial functional mitral regurgitation and atrial fibrillation may steepen the slope by altering left-atrial pressure or stroke volume reserve.^93,94^ Exercise tricuspid annular plane systolic excursion/systolic pulmonary artery pressure (TAPSE/sPAP), a proposed measure of right ventricular–pulmonary arterial (RV–PA) coupling, may provide complementary or potentially simpler alternative information to the flow-corrected mPAP/CO slope.^95^

Patients with negative HFpEF scores but abnormal PAP/CO slopes on echocardiography are at increased HF-related risk compared with those with negative scores and normal pressures and exhibit comparable exercise limitation to patients classified as HFpEF by scoring systems. These patients may benefit from invasive haemodynamic evaluation to confirm HFpEF and exclude pulmonary vascular disease.^88^ When paired with CPET (CPETecho), exercise echocardiography captures both haemodynamic, pulmonary, and peripheral domains in a single, simultaneous test, enhancing diagnostic precision and phenotypic resolution, and enabling dissection of Fick component variables of pVO_2_. Non-invasively, peripheral oxygen extraction (Ca–vO_2_) can be estimated by dividing VO_2_ from breath-by-breath gas exchange measurements by CO derived from echocardiography (a–vO_2_Diff = VO_2_/CO).^96^ This integrated CPETecho approach helps distinguish between central (cardiac output–limited) and peripheral (oxygen extraction–limited) contributors to EI, particularly when CO/VO_2_ is derived, with more shallow slopes implicating greater relative cardiac impairment. However, Ca–vO_2_ reflects the net result of both propensity for oxygen diffusion into peripheral tissues as well as capillary transit time, which determines the duration available for oxygen diffusion across the microcirculation. At higher cardiac output, reduced capillary transit time may limit oxygen extraction despite preserved mitochondrial oxidative capacity and peripheral diffusion capacity (DmO_2_).

##### Invasive haemodynamics

Use of high-fidelity micromanometer catheters with pressure–volume loop analysis permits quantification of the time constant of left ventricular relaxation (Tau, τ), along with the slope of the end-diastolic PV relationship reflecting LV stiffness (*b*). Criteria for HFpEF diagnosis include Tau > 48 ms or *b* > 0.27.^97^ Abnormalities in these invasively derived parameters were recently found to be highly prevalent in patients undergoing evaluation of exertional dyspnoea in a single-centre study,^98^ highlighting their potential clinical relevance for early detection of HFpEF. While Tau and LV stiffness represent principal pathophysiologic underpinnings of HFpEF, their assessment requires specialized equipment and expertise that are not routinely available in clinical practice.

Efforts to simplify ascertainment of these important parameters in the context of evaluating EI in suspected HFpEF, such as echocardiographic diastolic pressure volume quotient or single beat-derived stiffness parameters, warrant further investigation,^99,100^ and the downstream consequences of LV pressure elevation relative to CO augmentation during exercise are detailed below.

In the study by Reddy *et al*., application of the PCWP ≥ 25 mmHg vs PCWP/CO slope criterion (>2 mmHg/L/min) reclassified approximately 20% of patients with unexplained dyspnoea.^101^ Among those reclassified from HFpEF to control based on slope alone, elevated resting PCWP was more common, and CO augmentation during exercise was more robust.^101^ However, HF outcomes were not assessed to ascertain prognostic implications of reclassification in this study.^101^

**Figure 7:**
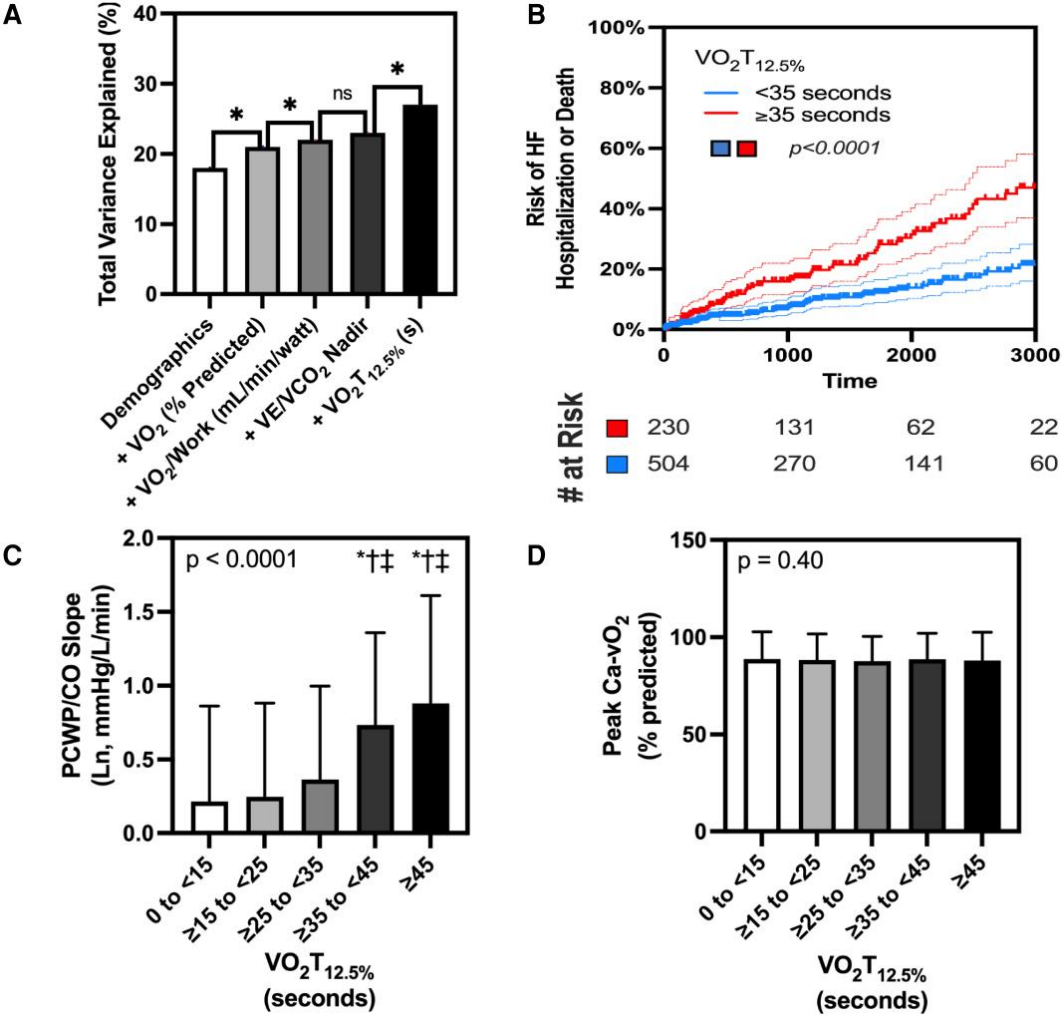
VO_2_T_12.5%_ vs non-invasive CPET measure in relation to the diagnosis and prognosis of HFpEF. (*A*) Demographics (age, sex, and body mass index) and other non-invasive cardiopulmonary exercise testing measures explained 23% of the variance in PCWP/CO slope. Addition of VO_2_T_12.5%_ improves the haemodynamic models to explain 27% of the total variance. *P < .05 vs both 0 to 15 s and 15 to 25 s, †*P* < .05 vs 25 to 35 s, ‡*P* < .05 vs 45 to 200 s. (*B*) Time to recover 12.5% of peak VO_2_ with a cut point of 35 s identified 230 patients at elevated risk of heart failure hospitalization and all-cause death. (*C*) Patients with a VO_2_T_12.5%_ ≥ 35 s had higher PCWP/CO slope than VO_2_T_12.5%_ < 35 s. *P < .05 vs VO_2_T_12.5%_ in DeLong test. (*D*) There was no difference in per cent predicted peak peripheral extraction (peak peripheral extraction corrected for haemoglobin concentration) between groups. *Adapted from Campain et al with permission.*^102^ Abbreviations: Ca-vO_2_, peripheral oxygen extraction; CO, cardiac output; PCWP, pulmonary capillary wedge pressure; VO_2_T_12.5%_, time to oxygen uptake by 12.5%; and VO_2_, oxygen uptake

In a meta-analysis by Baratto *et al*., including 27 studies, PCWP/CO slopes in HFpEF cohorts subjected to both upright and supine exercise were consistently above the 2 mmHg/L/min threshold, whereas control cohorts demonstrated slopes below 2 mmHg/L/min (*Figure 2C*).^36,102^ In a large cohort of 814 patients with dyspnoea on exertion, a prolonged VO_2_T_12.5%_ was associated with elevated PCWP/CO slope, more so than other CPET variables, and notably was not related to peripheral oxygen extraction, underscoring its cardiac specificity (*Figure 7*).^102^ Importantly, VO_2_T_12.5%_ also independently predicted HF hospitalization and mortality.^102^ These findings support VO_2_T_12.5%_ as a practical non-invasive marker that captures cardiac limitations during exercise and merit integration into CPET interpretation frameworks when evaluating patients with suspected HFpEF with predominantly cardiac limitations.

##### Cardiopulmonary exercise testing with invasive haemodynamics (iCPET)

iCPET is the gold standard for mechanistic profiling and is particularly useful in cases with discordant or inconclusive non-invasive findings.^11,35,77,103^ It enables direct, repeated measurement of CO, PCWP, mean PAP, and peripheral oxygen utilization during exercise. Importantly, it has been shown to be safe, feasible, and reproducible, enabling comprehensive haemodynamic assessment without compromising maximal exercise capacity.^104^

Singular measures of haemodynamics in a state of rest are known to have limitations, particularly after prolonged overnight fasting that often precedes cardiac catheterization procedures and may result in patients being intravascularly more volume-depleted than usual, with resultant lower PCWP that falls below the diagnostic threshold for HFpEF of PCWP ≥15 mmHg. Conversely, dynamic assessment using matched measurements of PCWP and directly measured Fick CO—used to calculate the PCWP/CO pressure–flow relationship—enables higher-resolution physiologic phenotyping.^10,11,105^

Exercise slope-based indices, such as a PCWP/CO slope >2 mmHg/L/min and PAP/CO slope >3 mmHg/L/min, provide robust, effort-independent assessments of circulatory reserve in HFpEF (*Figure 2*).^11,103^ By capturing the dynamic relationship between pressure and flow throughout exercise, these metrics are independent of accurate transducer levelling and offer physiologic context beyond isolated absolute values.^10^ In contrast, fixed thresholds—such as a peak PCWP ≥25 mmHg or mPAP ≥30 mmHg may be exceeded at high workloads in normal individuals or in the setting of large respiratory swings.^106,107^ Baratto *et al* found that PCWP/CO slope >2 mmHg consistently differentiated HFpEF from control subjects across 27 studies,^36^ and PAP/CO slope >3 mmHg/L/min currently defines abnormal and predicts prognosis in patients with exertional dyspnoea (*Figure 2C*).^103,108^ For upright iCPET, PAP/CO and PCWP/CO slopes have been shown to potently predict outcomes independent of resting pressures (Supplementary data online, *Table S1* and *Figure 2E*).^10,109^ For supine iCPET, PCWP indexed to workload, absolute PCWP ≥ 25 mmHg, and PCWP/CO slope have all been shown to predict prognosis across studies comprised >3000 HFpEF patients to date. The establishment of strong associations between exercise haemodynamic measures and HF outcomes lends validity to the incorporation of exercise haemodynamic measurements into diagnostic algorithms for HFpEF.

Body position during exercise testing also influences diagnostic accuracy. Recent findings by Fudim *et al.* demonstrate that half of the patients who met HFpEF criteria in the supine position (using exPCWP ≥ 25 mmHg, which was also used to anchor HF diagnostic scores) did not meet criteria during upright exercise (i.e. PCWP/CO slope > 2 mmHg/L/min).^77^ These discordant patients were exclusively re-classified from meeting criteria for HFpEF while supine but not upright, whereas all patients with abnormal upright haemodynamic response to exercise had abnormal supine exercise haemodynamic responses.^77^ Importantly, patients only meeting supine exercise haemodynamic criteria but not upright criteria had fewer structural and haemodynamic abnormalities, fewer common underlying conditions associated with HFpEF (such as atrial fibrillation), and lower H_2_FPEF scores,^4^ suggesting milder central haemodynamic impairment during upright exercise and highlighting the need to look beyond left heart filling pressures alone to understand exercise limitations.

#### Peripheral (muscle) domain

A major advantage of iCPET is the ability to simultaneously directly measure Fick CO and peripheral oxygen extraction through measurement of arterial and (mixed) venous blood gases to calculate the arteriovenous oxygen content difference (Ca–vO_2_).^6,110^ By plotting CO relative to VO_2_ throughout exercise, the relative predominance of deficits in CO vs peripheral extraction is evident by the slope of this relationship, which is normally approximately 5–6 litres of CO per 1 litre augmentation in VO_2_.^111^ Lower CO slopes indicate disproportionate reliance on widening peripheral O_2_ extraction to augment VO_2,_ whereas higher CO values indicate greater impairment in peripheral O_2_ extraction rather than CO during exercise (*Figure 4*).

This elegant approach to defining relative impairments revealed that reduced CO compared with expected in HFrEF predicts cardiovascular (CV) events, whereas steeper slopes in a subset of HFrEF patients signalled more peripheral impairment and fewer CV events.^111^ Further study is needed to determine the extent to which greater relative cardiac impairment in HFpEF leads to higher CV event ranges. One study with direct central and peripheral measures of convective delivery and diffusive conductance of O_2_, respectively, in HFpEF and HFrEF found greater impairment in diffusive conductance of O_2_ in HFpEF compared with HFrEF and thereby suggested greater VO_2_ improvement with correction of peripheral oxygen utilization as opposed to central haemodynamics (*Figure 4D*, **black and white arrows**).^58^

#### Pulmonary (lung) domain

Gas exchange patterns during exercise provide a window into abnormalities in pulmonary parenchymal and vascular function in HFpEF. During upright exercise, overt hypoxaemia is uncommon in the setting of HFpEF with isolated elevation in PCWP, whereas oxygen saturation and partial pressure of oxygen (PaO_2_) inversely correlate with exercise pulmonary vascular resistance (PVR), and therefore desaturation should prompt consideration of abnormal pulmonary vascular function in HFpEF.^112–115^ In HFpEF patients undergoing supine exercise, Omar *et al.* found O_2_ desaturation to be more common with both elevated PAP and PCWP.^113^ Similarly, steep VE/VCO_2_ relationships (i.e. >36) reflect ventilation/perfusion (V/Q) mismatch and heightened fractional dead space that makes pulmonary vascular dysfunction more likely and is associated with adverse CV events (Supplementary data online, *Table S1*).

Elevated VE/VCO_2_ slope reflects ventilatory inefficiency, which is mechanistically linked to ventilation–perfusion mismatch, increased physiologic dead space, and abnormal pulmonary arterial coupling. Multiple studies have demonstrated that steeper VE/VCO_2_ slopes are independently associated with adverse outcomes in HFpEF, including incident HF events, all-cause mortality, and HF hospitalization.^115,116^ While the prognostic significance of ventilatory inefficiency in HFpEF is well established, the optimal cutoff for risk stratification remains less clearly defined than in HFrEF, where a VE/VCO_2_ slope >36 has been consistently validated as a threshold for poor prognosis.^117^ In HFpEF, lower cutoffs have been described, with Guazzi *et al*. identifying a threshold near 33 that predicted adverse outcomes,^118^ while other studies reported higher cutoffs.^119^ Thus, although a VE/VCO_2_ slope >36 can be pragmatically extrapolated from HFrEF, the optimal threshold in HFpEF has not been uniformly validated, and further studies are required to establish HFpEF-specific cutoffs.

In a study by Nayor *et al*., VE/VCO_2_ closely reflected invasively derived haemodynamic measures.^115^ Higher VE/VCO_2_, particularly at its nadir, was associated with lower peak CO, a steeper PCWP/CO slope, and increased risk of cardiovascular hospitalization or death.^115^ The authors further demonstrated that the method used to quantify VE/VCO_2_ can influence its physiologic and prognostic interpretation.^1^ Across both community and referral cohorts, VE/VCO_2_ behaviour varied with exercise intensity, with the nadir providing the most robust signal by minimizing the confounding effects of early or late hyperventilation.^115^

Understanding relative limitations imposed by pulmonary mechanics and the cardiovascular system is also of utility in HFpEF evaluation. A high ratio between V_E_ and maximal voluntary ventilation (MVV), reduced inspiratory capacity, or a post-exercise drop in forced expiratory volume in one second (FEV_1_) may indicate pulmonary mechanical restraints on exercise, particularly if VE/MVV exceeds 70% prior to reaching the ventilatory anaerobic threshold.^120^ Flow–volume loops obtained during exercise—particularly when combined with a maximal inspiratory manoeuvre—enhance diagnostic accuracy beyond resting spirometry. A tidal volume to inspiratory capacity ratio (TV/IC) > 0.9 is consistent with dynamic hyperinflation.^121^

#### Integrated approach: the HFpEF/dyspnoea clinic model

Dedicated HFpEF/dyspnoea clinics enable evaluations that integrate clinical data, biomarkers, echocardiography, lung function, and exercise testing with appropriately parsimonious deployment of more complex forms of evaluation such as invasive CPET (*Figure 6*). The growing experience with CPETecho to derive patterns defined in expert centres that utilize routing iCPET (i.e. pressure flow relationships and CO/VO_2_ slopes) will aid in more widespread exercise-based phenotyping of HFpEF, particularly if exercise-based subphenotypes are proven to respond to targeted interventions, as discussed below.

## Improving exercise capacity in HFpEF: current approaches and emerging therapies

**Figure 8:**
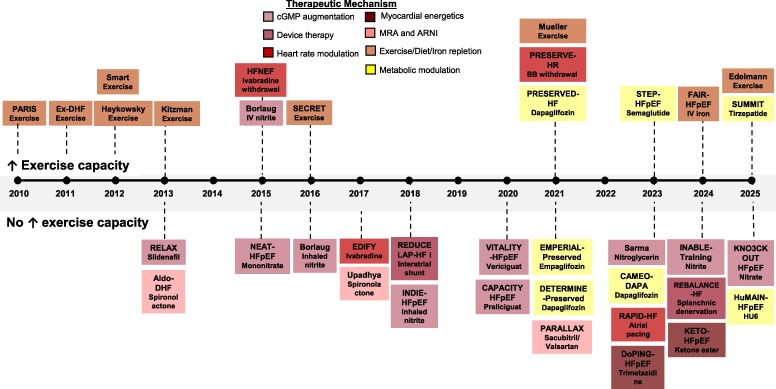

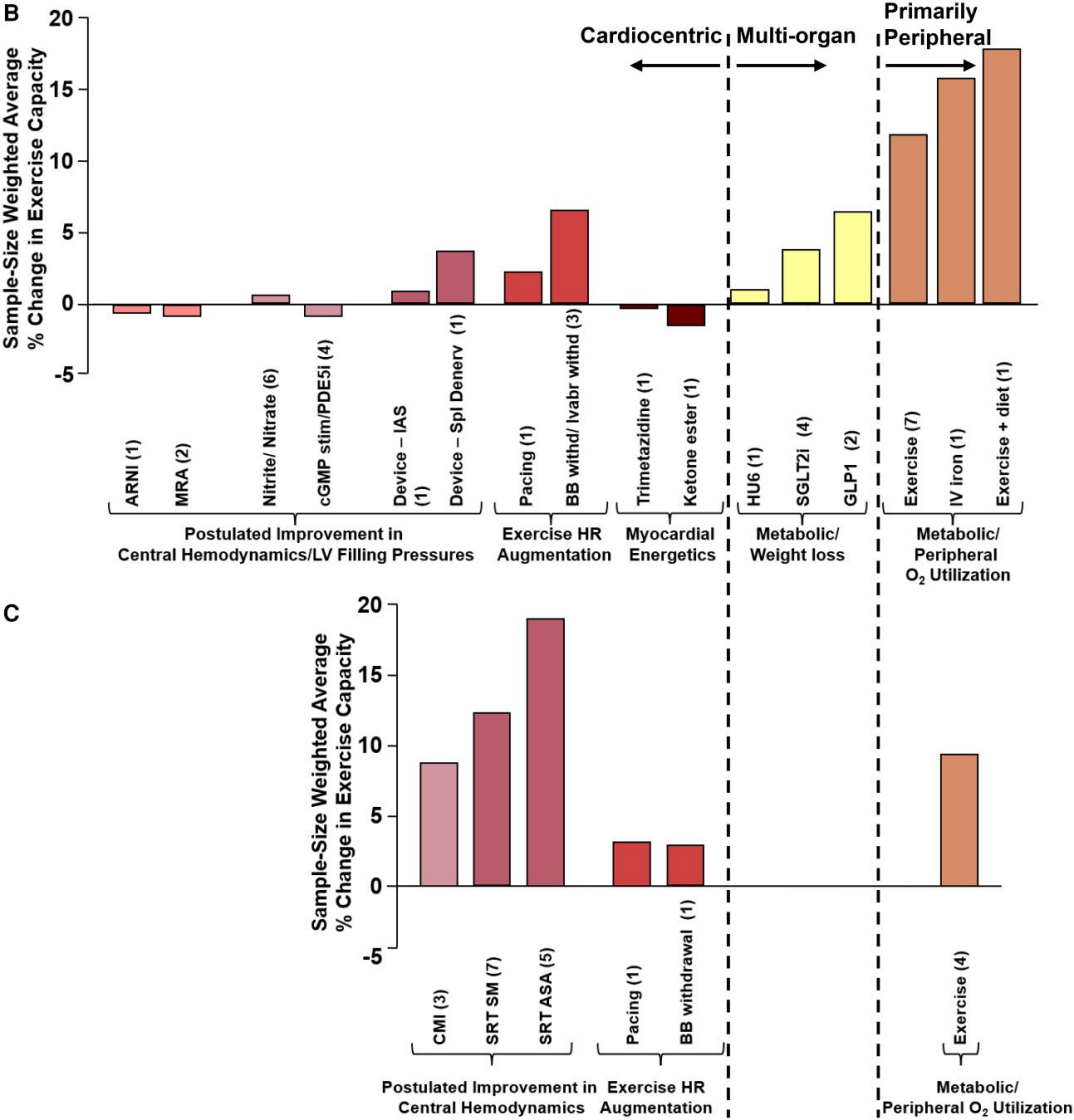

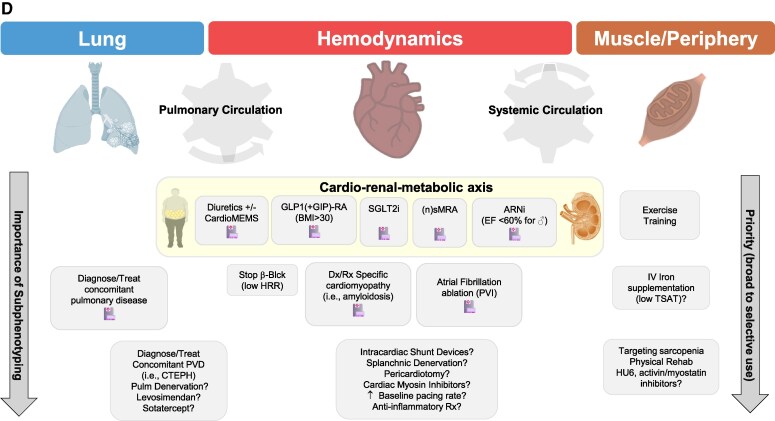
Therapeutic landscape and pathophysiological targets in HFpEF. (*A*) Timeline of randomized clinical trials in HFpEF over the past 15 years that reported exercise outcomes (peak VO_2_ or 6-minute walk distance) and had placebo, sham, or crossover-controlled designs. Trials positioned above the timeline demonstrated a statistically significant improvement in exercise capacity, whereas those below did not. Trials are colour-coded by therapeutic mechanism: cGMP augmentation, mineralocorticoid receptor antagonism (MRA) and angiotensin receptor-neprilysin inhibitor (ARNI), device therapy, exercise/diet/iron repletion, heart rate modulation, myocardial energetics, and metabolic modulation. (*B*) Sample size–weighted average percentage change in exercise capacity (peak VO_2_ or 6-minute walk distance) in HFpEF across therapeutic classes, grouped by hypothesized mechanism of benefit. Numbers in parentheses indicate the number of trials included for each intervention category. (*C*) Sample size–weighted average percentage change in exercise capacity (peak VO_2_) in hypertrophic cardiomyopathy across therapeutic classes, grouped by hypothesized mechanism of benefit. Numbers in parentheses indicate the number of trials included for each intervention category. (*D*) Conceptual model linking key pathophysiologic domains in HFpEF—pulmonary, haemodynamic, and peripheral—with therapeutic targets. Subphenotyping based on these domains may guide treatment, from broadly applicable therapies (e.g. SGLT2 inhibitors, exercise training) to more selective or investigational interventions (e.g. splanchnic denervation, myostatin inhibition). Abbreviations: ARNi, angiotensin receptor-neprilysin inhibitor; BMI, body mass index; CardioMEMS, wireless pulmonary artery pressure monitoring system; BB, beta-blocker; cGMP, cyclic guanosine monophosphate; CTEPH, chronic thromboembolic pulmonary hypertension; Dx/Rx, diagnosis/treatment; EF, ejection fraction; GIP, glucose-dependent insulinotropic polypeptide; GLP1, glucagon-like peptide-1 receptor agonist; HR, heart rate; Ivabr; ivabradine; IV, intravenous; LV, left ventricle; IAS, interatrial shunt; MRA, mineralocorticoid receptor antagonist; PDE5i, phosphodiesterase type 5 inhibitor; PVD, pulmonary vascular disease; SGLT2i, sodium-glucose cotransporter-2 inhibitor; Spl Denerv, splanchnic denervation; withdr, ivabradine withdrawal

A broad range of lifestyle, pharmacological, and device interventions has been evaluated in clinical trials with exercise endpoints (*Figure 8A*) and (*Table 1*),^21,24,122–163^ and the collective results offer insights into predominant mechanisms governing EI in HFpEF (*Figure 8B*–*D*).

**Table 1:** Randomized, placebo-, sham-controlled, or crossover trials in HFpEF published in the last 15 years that included objective exercise measurement as endpoints

Clinical trial	Design	N subjects	Intervention	Study population	Exercise endpoints	Findings related to exercise haemodynamic measurements	Impact on exercise capacity
**Device/Procedure interventions**							
**Intra-atrial shunt**							
Feldman *et al*, 2018^122^ (REDUCE-LAP HF I, NCT02600234)	Phase 2, randomized, multicentre, sham-controlled, parallel-group	44	Interatrial shunt	HFmEF & HFpEF LVEF ≥ 40% and exercise PCWP ≥ 25 mmHg while exceeding RAP by ≥ 5 mmHg	Primary: supine exercise PCWP at 1 month Secondary: Δ PCWP at rest, legs up, 20 watts, and peak exercise	Peak exercise PCWP decreased 3.5 ± 6.4 mmHg with shunt vs 0.5 ± 5.0 mmHg in the control group (*P* = .14)	No significant Δ exercise capacity: peak supine workload increased 1.5 ± 14.6 watts in shunt vs −1.9 ± 10.8 watts in controls (*P* = .35); and time increased 1.2 ± 3.7 min with shunt vs 0.4 ± 3.5 min in controls (*P* = .60)
**Splanchnic denervation**							
Fudim *et al*, 2024^123^ (REBALANCE-HF, NCT04592445)	Phase 2, randomized, double-blind, multicentre, sham-controlled	90	Splanchnic Denervation	HFpEF (EF ≥ 40%) and exercise PCWP ≥ 25 mmHg	Primary: Δ PCWP with legs up and at 20 watts of exercise Secondary: Δ 6MWD at 3, 6, and 12 months	No change in primary endpoint with mean between-group difference in PCWP of −0.03 mmHg (95% CI, −2.5 to 2.5; *P* = .95)	No difference in Δ 6MWD at 3 months (−4.2; 95% CI −27.5 to 19.1; *P* = .72), 6 months (7.1; 95% CI −20.5 to 34.6; *P* = .61), or 12 months (11.1; 95% CI −19.1 to 41.4; *P* = .47)
**Pharmacological interventions**							
**Mineralocorticoid receptor antagonist**							
Edelmann *et al*, 2013^124^ (Aldo-DHF, ISRCTN94726526)	Multicentre, randomized, double-blind, placebo-controlled	422	Spironolactone	HFpEF (LVEF ≥50%), NYHA class II–III symptoms, and pVO_2_ ≤ 25 mL/kg/min	Primary: Δ pVO_2_ at 12 months	No invasive haemodynamics	No significant Δ pVO_2_ in spironolactone vs placebo (adjusted mean difference, +0.1 mL/min/kg; 95% CI, −0.6 to +0.8 mL/min/kg; *P* = .81)
Upadhya *et al*, 2017^125^ (no trial abbreviation, no NCT)	Single-centre, randomized, placebo-controlled, double-blind trial	71	Spironolactone	HFpEF (LVEF ≥50%)	Primary: Δ pVO_2_ at 9 months	No invasive haemodynamics	No significant Δ pVO_2_ in spironolactone vs placebo (adjusted mean difference −0.4 mL/kg/min; 95% CI, −1.1 to 0.4 mL/kg/min; *P* = .38)
**Angiotensin receptor-neprilysin inhibitor**							
Pieske *et al*, 2021^126^ (PARALLAX, NCT03066804)	Multicentre, randomized, double-blind, active-controlled	2566	Sacubitril/Valsartan	HFpEF (LVEF ≥40%) and NYHA class II–IV symptoms	Coprimary: Δ 6MWD at week 24	No invasive haemodynamics	Sacubitril/valsartan vs standard treatment with enalapril, valsartan, or placebo did not significantly change the 6MWD (adjusted mean difference, −2.5 m; 95% CI, −8.5 to 3.5; *P* = .42)
**cGMP augmentation**							
Redfield *et al*, 2013^127^ (RELAX, NCT00763867)	Phase 3, randomized, multicentre, double-blind, placebo-controlled, parallel-group	216	Sildenafil	HFpEF (LVEF ≥50%) and NYHA class II–IV symptoms	Primary: Δ pVO_2_ at week 24 Secondary: Δ 6MWD at week 12 and 24, Δ pVO_2_ at week 12	No invasive haemodynamics	Δ pVO_2_ at 24 weeks was not significantly different (mean difference between sildenafil and placebo of 0.01 mL/min/kg, favoring sildenafil; 95% CI, −0.60 to 0.61; *P* = .98). Δ 6MWD at 24 and 12 weeks and Δ pVO_2_ at 12 weeks were not significantly different between sildenafil vs Placebo
Borlaug *et al*, 2015^128^ (no trial abbreviation, NCT01932606)	Phase 2, randomized, single-centre, double-blind, placebo-controlled, parallel-group	28	Intravenous sodium nitrite	HFpEF (LVEF ≥50%) and increased left heart filling pressures (PCWP at rest >15 mmHg and/or with exercise ≥25 mmHg)	Primary: Δ PCWP at exercise Secondary: Δ VO_2_ at 20 watts, exercise changes in RAP, PA pressure, PVR, PA compliance, systemic BP, HR, SV, stroke work, CO, and Ca-vO_2_	Exercise PCWP was improved with nitrite infusion compared with placebo (adjusted mean 19 ± 5 mmHg vs 28 ± 6 mmHg; *P* = .0003)	Nitrite slightly increased the VO_2_ achieved at 20 W compared with placebo (*P* = .02)
Redfield *et al*, 2015^129^ (NEAT-HFpEF, NCT02053493)	Phase 2, randomized, multicentre, double-blind, placebo-controlled, crossover	110	Isosorbide mononitrate (dose-escalation regimen)	HFpEF (LVEF ≥50%) with exercise intolerance primarily due to dyspnoea, fatigue, or chest pain	Primary: Δ accelerometer units during the period patients were receiving 120 mg of isosorbide mononitrate Secondary: Δ 6MWD	No invasive haemodynamics	Nonsignificant trend toward lower daily activity in the mononitrate group (−381 accelerometer units; 95% CI, −780 to 17; *P* = .06). No significant between-group differences in 6MWD
Borlaug *et al*, 2016^130^ (no trial abbreviation, NCT02262078)	Phase 2, randomized, single-centre, double-blind, placebo-controlled, parallel-group	26	Inhaled sodium nitrite	HFpEF (LVEF ≥50%) and increased left heart filling pressures (PCWP at rest >15 mmHg and/or with exercise ≥25 mmHg)	Primary: Δ PCWP at exercise Secondary: Δ VO_2_ at 20 watts, exercise changes in RAP, PA pressure, PVR, PA compliance, systemic BP, HR, SV, stroke work, CO, and Ca-vO_2_	Exercise PCWP was improved by nitrite as compared with placebo (baseline-adjusted mean 25 ± 5 vs 31 ± 6 mmHg; analysis of covariance *P* = .022)	There was no statistically significant effect of nitrite on VO_2_ at 20 watts compared with placebo
Borlaug *et al*, 2018^131^ (INDIE-HFpEF, NCT02742129)	Phase 2, randomized, multicentre, double-blind, placebo-controlled, crossover	98	Inhaled inorganic nitrite	HFpEF (LVEF ≥50%) with exercise intolerance primarily due to dyspnoea, fatigue, or chest pain	Primary: Δ pVO_2_ at week 4 Secondary: Δ accelerometer units	No invasive haemodynamics	No significant Δ pVO_2_ as compared with placebo (treatment effect of −0.20 mL/kg/min; 95% CI, −0.56 to 0.16; *P* = .27). No significant effect on daily activity levels (5497 vs 5503 accelerometry units per day; difference, −15; 95% CI, −264 to 234; *P* = .91)
Armstrong *et al*, 2020^132^ (VITALITY-HFpEF, NCT03547583)	Phase 2b, randomized, multicentre, double-blind, placebo-controlled	789	Vericiguat	HFpEF (LVEF ≥45%) and NYHA class II–III symptoms	Secondary: Δ 6MWD at week 24	No invasive haemodynamics	The least-squares mean difference in 6MWD between the 15-mg/d vericiguat and placebo was −5.5 m (95% CI, −19.7 m to 8.8 m; *P* = .45) and between the 10-mg/d vericiguat and placebo was −1.8 m (95% CI, −16.2 m to 12.6 m; *P* = .81)
Udelson *et al*, 2020^133^ (CAPACITY HFpEF, NCT03254485)	Phase 2, randomized, multicentre, double-blind, placebo-controlled	181	Praliciguat	HFpEF (LVEF ≥40%), NYHA class II–IV symptoms, impaired pVO_2_, and at least 2 conditions associated with nitric oxide deficiency	Primary: Δ pVO_2_ at week 12 Secondary: Δ 6MWD and Δ VE/VCO_2_ slope at week 12	No invasive haemodynamics	Placebo-adjusted least-squares between-group difference in pVO_2_ mean change from baseline was −0.30 mL/kg/min (95% CI, −0.95 to 0.35; *P* = .37), in 6MWD was −16.7 m (95% CI, −47.4 to 13.9), and VE/VCO_2_ slope was −0.3 (95% CI, −1.6 to 1.0)
Sarma *et al*, 2023^134^ (no trial abbreviation, NCT04068844)	Single centre, single-blind, crossover	30	Sublingual nitroglycerine (SL NTG)	HFpEF (LVEF >50%) and objective evidence of volume overload	Primary: Δ PCWP and pVO_2_ at rest, 20 watts, and peak exercise	At peak exercise, SL NTG decreased PCWP by 7 ± 6 mmHg (*P* = .004)	There was no statistical difference in pVO_2_ with SL-NTG vs placebo
Borlaug *et al*, 2024^135^ (INABLE-Training, NCT02713126)	Phase 4, randomized, multicentre, double-blind, placebo-controlled	73	Exercise training and inhaled nitrite	HFpEF (LVEF ≥50%) and NYHA class II–IV symptoms	Primary: Δ pVO_2_ at week 12 Secondary: Δ 6MWD at week 12	No invasive haemodynamics	Exercise training improved pVO_2_ (+0.8 mL/kg/min; 95% CI:0.3 to 1.2; *P* < .001), but nitrite did not increase pVO_2_ (nitrite effect −0.13; 95% CI−1.03 to 0.76; *P* = .77) or 6MWD (+7 m; 95% CI −13 to 26; *P* = .50)
Zamani *et al*, 2025^136^ (KNO3CK OUT HFpEF, NCT02840799)	Phase 2, randomized, multicentre, double-blind, crossover	84	Potassium nitrate (KNO3)	HFpEF (LVEF >50%) and evidence of elevated intracardiac filling pressures	Coprimary: Δ pVO_2_ and total work performed at week 6	No invasive haemodynamics	KNO3 did not improve pVO_2_ (0.06; 95% CI −0.32 to 0.45 mL/kg/min; *P* = .73) or total work performed (2.27; 95% CI −1.98 to 6.51 kJ; *P* = .29)
**Heart rate modulation**							
Pal *et al*, 2015^137^ (HFNEF, NCT02354573)	Single-centre, randomized, crossover, placebo-controlled	22	Ivabradine	HFpEF (LVEF ≥50%) and pVO_2_ < 80% of predicted	Primary: Δ pVO_2_ at week 2	No invasive haemodynamics	Δ pVO_2_ decreased in the ivabradine group (−2.1 vs 0.9 mL/kg/min; *P* = .003)
Komajda *et al*, 2017^138^ (EDIFY, no NCT)	Phase 2, randomized, multicentre, double-blind, placebo-controlled	179	Ivabradine	HFpEF (LVEF ≥45%)	Coprimary: Δ 6MWD at 8 months	No invasive haemodynamics	No improvement in Δ 6MWD (between-group estimate −3.8; IQR −19.1 to 11.6; *P* = .8)
Palau *et al*, 2021^139^ (PRESERVE-HR, NCT03871803)	Phase 4, randomized, single-centre, crossover	52	Beta-blocker withdrawal	HFpEF (LVEF >50%) and NYHA class II–IV symptoms	Primary: Δ pVO_2_ at end of trial	No invasive haemodynamics	pVO_2_ increased significantly after beta-blocker withdrawal (+2.1 ± 1.29 mL/kg/min; *P <* .001)
Reddy *et al*, 2023^140^ (RAPID-HF, NCT02145351)	Randomized, single-centre, double-blind, crossover	29	Pacemaker with atrial rate-responsive pacing	HFpEF (LVEF ≥40%) and NYHA class II–IV symptoms	Primary: Δ VO_2_ at anaerobic threshold at week 4 Secondary: Δ pVO_2_ and Δ VE/VCO_2_ slope at week 4	No invasive haemodynamics	No significant effect of pacing on VO_2_ at anaerobic threshold (mean difference 0.3 mL/kg/min; 95% CI −0.5 to 1.0 mL/kg/min; *P* = .46), Δ pVO_2_ (mean difference 0.4 mL/kg/min; 95% CI −0.4 to 1.2 mL/kg/min; *P* = .27), or Δ VE/VCO_2_ (mean difference 0.5; 95% CI, −0.6 to 1.6; *P* = .34)
**SGLT2 inhibitors**							
Nassif *et al*, 2021^141^ (PRESERVED-HF, NCT03030235)	Phase 4, randomized, multicentre, double-blind, placebo-controlled	324	Dapagliflozin	HFpEF (LVEF ≥45%) and NYHA class II–IV symptoms	Secondary: Δ 6MWD at week 12	No invasive haemodynamics	Improvement in 6MWD with mean effect size 20.1 m (95% CI 5.6 to 34.7, *P* = .007)
Abraham *et al*, 2021^142^ (EMPERIAL-Preserved, NCT03448406)	Phase 3, randomized, multicentre, double-blind, placebo-controlled	312	Empaglifozin	HFpEF (LVEF ≥40%)	Primary: Δ 6MWD at week 12	No invasive haemodynamics	Median difference in 6MWD between placebo and empaglifozin was 4.0 m (95% CI −5.0 to 13.0; *P* = .37)
Borlaug *et al*, 2023^143^ (CAMEO-DAPA, NCT04730947)	Phase 2, randomized, single-centre, double-blinded, placebo-controlled	37	Dapagliflozin	HFpEF (LVEF ≥50%) and NYHA class II–IV symptoms	Primary: Δ PCWP at week 24 Secondary: Δ RAP, PAP, pVO_2,_ and Ca-vO_2_	Exercise PCWP decreased with dapaglifozin treatment (−5.7 mmHg; 95% CI, −10.8 to −0.7; *P* = .027)	No differences in pVO_2_ at 20 watts or peak exercise
McMurray *et al*, 2024^144^ (DETERMINE-Preserved, NCT03877224)	Phase 3, randomized, multicentre, double-blind, placebo-controlled	253	Dapagliflozin	HFpEF (LVEF ≥40%)	Coprimary: Δ 6MWD at week 16	No invasive haemodynamics	No significant difference in 6MWD (median difference was 1.6 meters; 95% CI, −5.9, 9.0; *P* = .67)
**GLP-1 agonist**							
Kosiborod *et al*, 2023^145^ (STEP-HFpEF, NCT04788511)	Phase 3, randomized, multicentre, double-blind, placebo-controlled	529	Semaglutide	HFpEF (LVEF ≥50%) and BMI ≥30 kg/m^2^	Secondary: Δ 6MWD at week 52	No invasive haemodynamics	Δ 6MWD was 21.5 m in the semaglutide group and 1.2 m in the placebo group (estimated difference, 20.3 m; 95% CI, 8.6 to 32.1; *P* < .001)
Packer *et al*, 2025^146^ (SUMMIT, NCT04847557)	Phase 3, randomized, multicentre, double-blind, placebo-controlled	731	Tirzepatide	HFpEF (LVEF ≥50%) and BMI ≥30 kg/m^2^	Secondary: Δ 6MWD at week 52	No invasive haemodynamics	Δ 6MWD was 26.0 m in the tirzepatide group and 10.1 m in the placebo group (between-group median difference, 18.3; 95% CI, 9.9 to 26.7; *P* < .001)
**Metabolic accelerator**							
Pandey *et al*, 2025^147^ (HuMAIN-HFpEF, NCT05284617)	Phase 2a, randomized, multicentre, double-blind, placebo-controlled	66	HU6 (dose escalation)	HFpEF (LVEF ≥50%) and BMI ≥30 kg/m^2^	Secondary: Δ pVO_2_ and Δ 6MWD at week 19	No invasive haemodynamics	Nonsignificant between-group difference in pVO_2_ 0.13 (95% CI, −0.73 to 0.99; P = NS) and in 6MWT 12.6 (95% CI, −27.4 to 52.6, P = NS)
**Intravenous iron**							
Von Haehling *et al*, 2024^148^ (FAIR-HFpEF, NCT03074591)	Phase 4, randomized, multicentre, double-blind, placebo-controlled	39	Intravenous ferric carboxymaltose	HFpEF and iron deficiency	Primary: Δ 6MWD at week 24	No invasive haemodynamics	Δ 6MWD was greater for those who received iron compared with placebo (least square mean difference 49 m; 95% CI, 5 to 93; *P* = .029)
Lewis *et al*, 2025 (IRONMET-HFpEF, NCT04945707)	Phase 4, single-centre, double-blind	66	Intravenous ferric derisomaltose	HFpEF and iron deficiency	Primary: Δ pVO_2_ at week 12	No invasive haemodynamics	Ongoing
**Exercise training**							
**Exercise**							
Kitzman *et al*, 2010^149^ (PARIS, NCT01113840)	Single-centre, randomized, attention-controlled, single-blind study	53	Exercise training	HFpEF (LVEF ≥50%)	Primary: Δ pVO_2_ at week 16	No invasive haemodynamics	Peak VO_2_ significantly increased in the exercise training vs control group (change 2.3 ± 2.2 mL/kg/min vs −0.3 ± 2.1 mL/kg/min; *P* = .0002)
Edelmann *et al*, 2011^150^ (Ex-DHF, ISRCTN86879094)	Multicentre, randomized, single-blind	64	Exercise training	HFpEF (LVEF ≥50%) and NYHA class II–III symptoms	Primary: Δ pVO_2_ at 3 months	No invasive haemodynamics	Peak VO_2_ significantly increased in the exercise training vs control group (mean benefit of exercise training was 3.3 mL/min/kg; 95% CI, 1.8 to 4.8; *P* < .001)
Smart *et al*, 2012^151^ (no trial abbreviation, no NCT)	Single-centre, randomized, single-blind	25	Exercise training	HFpEF (LVEF ≥45%)	Primary: Δ pVO_2_ at week 16	No invasive haemodynamics	After exercise training the increment in pVO_2_ in the exercise training group was (24.6%, *P* = .02) vs control group (5.1%, *P* = .19)
Haykowsky *et al*, 2012^152^ (PARIS, NCT01113840)	Single-centre, randomized, attention-controlled, single-blind study	40	Exercise training	HFpEF (LVEF ≥50%)	Primary: Δ pVO_2_ at 4 months	No invasive haemodynamics	Peak VO_2_ significantly increased in the exercise training vs control group (16.3 ± 2.6 mL/kg/min vs 13.1 ± 3.4 mL/kg/min; *P* = .002)
Kitzman *et al*, 2013^153^ (no trial abbreviation, no NCT)	Single-centre, randomized, single-blind	63	Exercise training	HFpEF (LVEF ≥50%)	Secondary: Δ pVO_2_ at week 16	No invasive haemodynamics	Peak VO_2_ significantly increased in the exercise training vs control group (15.8 ± 3.3 vs 13.8 ± 3.1 mL/kg/min, *P* = .0001)
Kitzman *et al*, 2016^154^ (SECRET, NCT00959660)	Single-centre, randomized, attention-controlled	100	Exercise training and diet	HFpEF (LVEF ≥50%) and BMI ≥30kg/m^2^	Primary: Δ pVO_2_ at week 20	No invasive haemodynamics	Peak VO_2_ was increased significantly by both exercise (main effect 1.2 mL/kg/min; 95%CI, 0.7 to 1.7; *P* < .001) and diet (main effect 1.3 mL/kg/min; 95%CI, 0.8 to 1.8; *P* < .001). The combination of Exercise and Diet was additive for pVO_2_ (joint effect 2.5 mL/kg/min)
Mueller *et al*, 2021^21^ (no trial abbreviation, NCT02078947)	Multicentre, randomized	180	High-intensity interval training, moderate continuous training	HFpEF (LVEF ≥50%) and sedentary	Primary: Δ pVO_2_ at 3 months	No invasive haemodynamics	Δ pVO_2_ for high-intensity interval training vs control was 1.1 vs −0.6 mL/kg/min (difference, 1.5; 95% CI, 0.4 to 2.7); for moderate continuous training vs control, 1.6 vs −0.6 mL/kg/min (difference, 2.0; 95% CI, 0.9 to 3.1); and for high-intensity interval training vs moderate continuous training, 1.1 vs 1.6 mL/kg/min (difference, −0.4; 95% CI, −1.4 to 0.6)
Edelmann *et al*, 2025^24^ (Ex-DHF, ISRCTN86879094)	Multicentre, randomized	322	Exercise training	HFpEF (LVEF ≥50%) and NYHA class II–III symptoms	Secondary: Δ pVO_2_ at 6 and12 months	No invasive haemodynamics	Δ pVO_2_ was significantly different between groups at 6 months and 12 months (mean differences of 0.8 mL/kg/min; 95% CI, 0.0 to 1.6, and 1.3 mL/kg/min; 95% CI, 0.4 to 2.1, respectively)
**Additional therapies** ^a^							
**Ketone ester**							
Selvaraj *et al*, 2025^155^ (NCT04633460)	Phase 2, randomized, single-centre, double-blind, crossover, placebo-controlled	20	Ketone ester	HFpEF (LVEF ≥50%)	Coprimary: Δ pVO_2_ and time to exhaustion during an additional constant-intensity exercise (75% peak workload). Secondary: echocardiographic assessments of diastolic function and stroke volume throughout stages of exercise	No invasive haemodynamics	No improvement in pVO_2_ (10.4 ± 3.6 vs placebo 10.5 ± 4.0 mL/kg/min; *P* = .75). No improvement in exercise endurance during the constant-intensity protocol
Gopalasingam *et al*, 2024^156^ (KETO-HFpEF, NCT05236335)	Randomized, double-blind, placebo-controlled crossover study	24	Ketone ester	HFpEF (LVEF >40%) and type 2 diabetes mellitus	Primary: CO during the 4-hour rest period after intake of ketone ester or placebo Secondary: PCWP, mPAP, SVR, pulmonary vascular resistance, and LVEF at rest, pressure-flow relationship ΔPCWP/ΔCO, exercise capacity	At peak exercise, ketone ester increased CO by 1.0 L/min (95% CI, −0.5 to 2.4) and decreased PCWP by 5 mmHg (95% CI, −9 to −1) compared with placebo. During the full exercise test, ketone ester decreased ΔPCWP/ΔCO by 0.2 mm Hg·L^−1^·min^−1^ (95% CI, −0.4 to 0.0)	There was no statistically significant difference in peak VO_2_ (*P* = .70)
**Verinurad plus allopurinol**							
Kitzman *et al*, 2024^157^ (AMETHYST, NCT04327024)	Phase 2, randomized, multicentre, double-blind	159	Verinurad and allopurinol or allopurinol monotherapy	HFpEF (LVEF ≥45%) and hyperuricemia	Primary: Δ pVO_2_ at week 32	No invasive haemodynamics	Δ pVO_2_ was similar across groups (verinurad plus allopurinol, 0.27 mL/kg/min; 95% CI, −0.56 to 1.10; allopurinol, −0.17 mL/kg/min; 95% CI, −1.03 to 0.69; placebo, 0.37 mL/kg/min; 95% CI, −0.45 to 1.19)
**Pirfenidone**							
Lewis *et al*, 2022^158^ (PIROUETTE, (NCT02932566)	Phase 2, randomized, multicentre, double-blind, placebo-controlled	80	Pirfenidone	HFpEF (LVEF ≥45%) and elevated natriuretic peptides	Secondary: Δ 6MWD at week 52	No invasive haemodynamics	Non-significant between-group difference in Δ 6MWD (15.54 m, 95% CI, −9.55 to 40.63)
**Adenosine agonist**							
Shah *et al*, 2019^159^ (PANACHE, NCT03098979)	Phase 2b, randomized, multicentre, double-blind, parallel-group	262	Neladenoson	HFpEF (LVEF ≥45%)	Primary: Δ 6MWD at week 20	No invasive haemodynamics	None of the neladenoson groups achieved the defined clinically relevant 40-m increase in 6MWD from baseline
**Levosimendan**							
Burkhoff *et al*, 2021^160^ (HELP, NCT03541603)	Phase 2, randomized, multicentre, double-blind, placebo-controlled	37	Levosimendan	PH-HFpEF (mPAP ≥35 mmHg and PCWP ≥20 mmHg) and NYHA class II–III symptoms	Primary: Δ PCWP at peak exercise at week 6 Secondary: Δ PCWP assessed across stages of exercise and Δ 6MWD	No significant reduction in peak PCWP in levosimendan compared with placebo group (−1.4 mmHg; 95% CI, −7.8 to 4.8; *P* = .65). Levosimendan reduced PCWP measured across all exercise stages (−3.9 ± 2.0 mm Hg; *P* = .047)	Levosimendan improved 6MWD by 29.3 m (95% CI, 2.5 to 56.1; *P* = .033) compared with placebo
Yaku *et al*, 2025^161^ (LEVEL, NCT05983250)	Phase 3, randomized, multicentre, double-blind, placebo-controlled	230	Levosimendan	PH-HFpEF (elevated mPAP, PCWP, and RAP at rest or with passive leg raise or with exercise)	Primary: Δ 6MWD at week 12	No invasive haemodynamics	Ongoing
**Myocardial Energetics**							
Bovenkamp *et al*, 2023^162^ (DoPING-HFpEF)	Phase II single-centre, double-blind, placebo-controlled, randomized cross-over trial	25	Trimetazidine	HFpEF (LVEF ≥50%) and NYHA class II–IV symptoms	Primary: Δ PCWP Secondary: Δ myocardial phosphocreatine/adenosine triphosphate	No effect of trimetazidine on Δ PCWP (mean change 0 [95% CI −2, 2] mmHg over multiple levels of exercise, *P* = .60)	No change by trimetazidine compared with placebo in 6MWD (mean change of −6 [95% CI −18, 7] m vs −5 [95% CI −22, 22] m)
**Myeloperoxidase inhibition**							
Popovic *et al*, 2025^163^ (NCT03611153)	Single-centre, double-blind, randomized, placebo-controlled, parallel group trial	30	Mitiperstat	HFpEF (resting PCWP ≥ 15 mmHg or exercise PCWP ≥ 25 mmHg and LVEF ≥50%)	Primary: PCWP during 20-W exercise workload Secondary: Δ resting PCWP; rest and exercise Δ PAP and other haemodynamic measures	Compared with placebo, mitiperstat treatment resulted in a higher PCWP during the second bout of exercise (11 ± 3 vs −4 ± 5 mmHg; *P* = .04).	There was no statistically significant difference in peak VO_2_ (*P* = .70)

^a^Studies conducted in populations other than primary HFpEF—such as those focused specifically on PH-HFpEF (e.g. trials of levosimendan)—as well as single small neutral studies of pharmacotherapies with limited sample size were not captured in the table.

6MWD, six-minute walk distance; BMI, body mass index; Ca-vO_2_, arteriovenous oxygen content difference; CI, confidence interval; CO, cardiac output; DBP, diastolic blood pressure; EF, ejection fraction; HF, heart failure; HFmEF, heart failure with mildly reduced ejection fraction; HFpEF, heart failure with preserved ejection fraction; HR, heart rate; LVEF, left ventricular ejection fraction; mPAP, mean pulmonary artery pressure; NYHA, New York Heart Association; NCT, National Clinical Trial identifier; pVO_2_, peak oxygen uptake; PAP, pulmonary artery pressure; PCWP, pulmonary capillary wedge pressure; PH-HFpEF, pulmonary hypertension with HFpEF; RAP, right atrial pressure; RCT, randomized controlled trial; SBP, systolic blood pressure; SL NTG, sublingual nitroglycerin; SV, stroke volume; VE/VCO_2_, minute ventilation and carbon dioxide production; VO_2_, oxygen uptake.

## Interventions for all patients with HFpEF

### Supervised exercise training (SET)

SET is the most widely investigated HFpEF intervention intended to improve exercise capacity. SET confers both etiologic and symptomatic treatment of HFpEF,^164^ and primarily boosts peripheral oxygen extraction by increasing capillary recruitment,^165^ exercise hyperaemia,^166^ and respiratory efficiency^167^ while reducing intramuscular adipose tissue.^168^ Changes in skeletal muscles in HFpEF are partially distinct from those reported with deconditioning.^169^ In SET trials to date, including the recently published Exercise training in Diastolic Heart Failure (Ex-DHF) trial, changes in diastolic function and other measures of cardiac structure and function are either absent or modest.^24^ Three meta-analyses have reported consistent pVO_2_ increases of 2.1–2.2 mL/kg/min,^164,170^ vs sedentary control (all *P* < .001).^171^ High-intensity or interval training may hasten pVO_2_ gains, whereas the addition of resistance training and respiratory muscle training,^172^ can confer additive salutary effects on exercise capacity in HFpEF. However, (i) limited provider engagement in promoting exercise training; (ii) lack of insurance coverage for rehabilitation; (iii) common neuromuscular limitations; and (iv) challenges with sustaining chronic unsupervised exercise training limit widespread implementation of SET.

## Cardio-renal-metabolic interventions in congested HFpEF

The following interventions have been studied in trials with entry criteria that enriched for heightened wall stress and congestion based on natriuretic-peptide level entry criteria significantly above requisite values specified by the universal definition of HF, coupled with evidence of structural heart disease.

### Sodium-glucose cotransporter-2 (SGLT2) inhibitors

SGLT2 inhibitors reduce HF admissions and CV death across the left ventricular ejection fraction (LVEF) spectrum in HF, yet their impact on exercise capacity in trials has been inconsistent. In PRESERVED-HF (Dapaglifozin in Preserved Ejection Fraction Heart Failure, *n* = 289, median BMI of 34.8 kg/m^2^), dapagliflozin conferred a 20.1-m placebo-corrected improvement in 6 min walk distance (6MWD). Notably, the observed benefit was independent from markers of congestion,^141^ but improvements were most pronounced in participants with diabetes and BMI >34.8 kg/m^2^.^173^ Disappointingly, the DETERMINE-Preserved (Dapaglifozin Effect on Exercise Capacity Using a 6-min Walk Test in Patients with HFpEF) trial (*n* = 504, median BMI = 28.7 kg/m^2^) and EMPEROR-preserved (Empaglifozin Outcome Trial in Patients with Chronic HFpEF) trial (*n* = 5,988, 66.8% had LVEF > 50%, median BMI = 29.8 kg/m^2^) trials showed an overall neutral effect on 6MWD.^17,144^ One meta-analysis of 8 studies with 2624 patients demonstrated only a minimal, but statistically significant difference of 6.7 m between SGLT2 inhibitors and placebo after resolving heterogeneity between the studies.^174^ The CAMEO-DAPA (Cardiac and Metabolic Effects of Dapaglifozin in HFpEF) trial (*n* = 38, average BMI 34.7 kg/m^2^) notably demonstrated a reduction in rest and exercise PCWP without an associated increment in pVO_2_, mirroring results with cyclic guanosine monophosphate (cGMP) augmentation strategies that lower filling pressures but do not augment pVO_2_.^175^

### Aldosterone antagonists

The Aldo-DHF (Aldosterone Receptor Blockade in Diastolic Heart Failure) trial (*n* = 422, mean age of 67 years) used CPET and 6MWT to objectively document exercise capacity after a year of spironolactone 25 mg or placebo. Peak VO_2_ did not change significantly (adjusted mean difference +0.1 mL/min/kg [95% CI −0.6 to +0.8 mL/kg/min], *P* = .81), and spironolactone reduced 6MWD by 15 m (95% CI −27 to −2 m, *P* = .03) with discordant improvement in E/e’, LV mass, and NT-proBNP levels.^124^ Although spironolactone improved pVO_2_ in one study,^176^ meta-analysis of three studies with 519 patients showed no significant differences in walk distance.^177^ To our knowledge, the effect of Finerenone on exercise capacity in HFpEF has not been reported.

### Angiotensin receptor blockade and neprilysin inhibition

The effect of Valsartan and Sacubitril or Valsartan alone on EI in HFpEF was evaluated in two randomized trials.^178^ Valsartan had no significant effect on gas exchange variables or 6MWD in a randomized trial of 152 hypertensive patients with HFpEF.^179,180^ More recently, 2572 patients with LVEF ≥40% were assigned to sacubitril/valsartan or standard treatment (predefined as enalapril, valsartan, or placebo stratum).^126^ Sacubitril/valsartan didn’t improve 6MWD at 24 weeks.^126^

### Loop diuretics

Loop diuretics are frequently used to mitigate congestion-dependent EI, but randomized controlled trials to definitively understand the impact of loop diuretics on exercise capacity in HFpEF have not been reported to our knowledge.

## Interventions for the obese subphenotype of HFpEF

### Glucagon-like peptide-1 (GLP-1) agonists

Long-acting incretin mimetics result in marked weight loss in HFpEF.^181^ In the STEP-HpEF (semaglutide in patients with HFpEF and obesity) trials, patients with HFpEF, obesity, symptoms, and LVEF > 45% experienced 14 to 20-m placebo-corrected increments in 6MWD.^145,181^ More recently, the SUMMIT (tirzepatide for HFpEF and obesity) trial investigated a dual-acting GLP-1 and glucose-dependent insulinotropic polypeptide receptor agonist, tirzepatide, in 731 patients with a mean BMI of 38.2 kg/m^2^. Patients treated with weekly tirzepatide were able to walk 18 m more on 6MWT after 1 year.^182^

In the STEP-HFpEF and the SUMMIT trials, functional improvement achieved by Semaglutide or Tirzepatide was associated with significant weight loss (10.7% and 11.6%, respectively, treatment vs placebo), with average relative improvements in 6MWD of only 6% and 6.3%, respectively.^145,182^ In contrast, diet alone and diet-and-exercise studies by Kitzman *et al*. in HFpEF showed 6.6% and 10% weight loss with 6.3% and 21.4% average improvements in 6MWD, reflecting greater increments in walking distance relative to weight loss with diet or diet and exercise interventions.^154^ The modest gains in 6MWD observed with GLP-1 receptor agonists may reflect lean muscle mass loss and the persistence of multi-domain physiologic limitations in HFpEF. However, in a non-randomized observational study of 61 patients with severe obesity (BMI 50.45 kg/m^2^), bariatric surgery was associated with a 23.4% improvement in 6MWD alongside 21.4% weight loss in three months after gastric bypass surgery.^183^ Notably, in STEP-HFpEF, improvements in 6MWD plateaued by ∼20 weeks despite continued weight loss, suggesting that early functional gains are not solely attributable to mechanical unloading.^145^ More modest increments in 6MWD with GLP-1 agonists may be attributable to loss in lean muscle mass or may follow the modest changes in exercise capacity with other interventions in HFpEF, owing to the compound physiologic deficits that must be overcome to improve exercise capacity.^6^

### Dietary interventions

Dietary interventions may mitigate EI, particularly in obese, malnourished, sarcopenic, or hypertensive individuals. In a randomized factorial trial,^154^ 100 HFpEF patients (age 67 years, BMI 39.3 kg/m^2^) were assigned to a hypocaloric diet (350–400 kcal/day intake deficit) daily, 60-min supervised exercise sessions three times weekly, both or no interventions. After 20 weeks, pVO_2_ increased by 1.2 mL/kg/min with exercise and 1.3 mL/kg/min with diet alone. The highest improvement in pVO_2_ of 2.5 mL/kg/min was accomplished when diet and exercise were combined (*Figure 8*).^154^

Salt restriction has been explored in congested HFpEF patients. In a small, non-randomized proof-of-concept study, 13 hypertensive patients with HFpEF (age 72 years, BMI 35.5 kg/m^2^) received a DASH diet (1150 mg Na/2100 kcal) for 21 days.^184^ The intervention resulted in minimal but significant weight loss (1.7 kg), improved systolic and diastolic blood pressure (155 to 138 mmHg, *P* = .02; 79 to 72 mmHg, *P* = .04, respectively), and increased 6MWD (313 to 337 m, *P* = .006).

## Interventions for iron and other nutrient deficiencies in HFpEF

### Iron repletion

Iron is an indispensable component of cellular respiration, which is deficient in >50% of HFpEF patients, based on definitions of iron deficiency used in HF trials.^62^ The FAIR-HFpEF (Ferric Carboxymaltose and Exercise Capacity in HFpEF) trial (*n* = 39 patients, stopped prematurely due to slow enrolment) demonstrated a 6MWD increase of 49 ± 22 m, after administration of intravenous iron carboxymaltose.^148^

### Vitamin supplementation

Water-soluble vitamin deficiencies (i.e. thiamine, vitamin C, and B12) tend to be exacerbated by loop diuretics^185^ and may contribute to fatigue and EI. Vitamin B12 deficiency often arises in elderly patients on plant-based diets, after bariatric surgery, or when using metformin or proton pump inhibitors, though there is a paucity of randomized clinical trial evidence supporting routine use of vitamin supplements to augment exercise capacity in HFpEF.^186^

## Interventions for the chronotropic incompetence HFpEF

### Heart rate modulation

Clinical trials have shed some light on the question of whether chronotropic incompetence during exercise in HFpEF is an important therapeutic target or if alternative aetiologies of EI simply limit ‘chronotropic access’. Depending on the definition used, chronotropic incompetence is present in 42% to 80% of patients with HFpEF.^34,187,188^ The RAPID-HF (Rate-Adaptive Atrial Pacing for HFpEF) trial was a randomized crossover trial in 29 patients with HFpEF, during which a piezoelectric accelerometer was adjusted to achieve atrial pacing HR augmentation during vigorous walking exercise, while the control group had the accelerometer turned off.^140^ After 4 weeks, the participants had a CPET with non-invasive CO measurement and were switched to opposite settings. Peak HR was modestly increased (14 bpm difference, 123 bpm when pacing, and 109 bpm with pacing off); however, pVO_2_ did not change (0.4 mL/kg/min difference, 16.5 vs 16.8 mL/kg/min, *P* = .4) because HR augmentation was opposed by a 24 mL average decrease in peak stroke volume (112 vs 88 mL, *P* = .02).^140^ In HFrEF, similarly neutral results^189–191^ or modest benefits have been observed.^192^ However, chronotropic incompetence is not ‘an all or nothing’ condition, and the advantages of rate-adaptive pacing might be limited to patients with the most severe chronotropic incompetence.^193,194^

The use of beta-blockers, ivabradine, and potentially other medications with strong negative chronotropic effects could worsen EI in HFpEF. HFpEF patients may be particularly vulnerable to bradycardia because they have impaired stroke volume and peripheral extraction during exercise.

The elegant crossover PRESERVE-HR (Effect of Beta-blocker Withdrawal on Functional Capacity in HFpEF) trial (52 patients, mean BMI 31 kg/m^2^, age 73 years) assigned patients with chronotropic incompetence to the sequence of beta-blocker (bisoprolol in 88.5%) withdrawal vs continuation, followed by the opposite intervention in 15 days.^139^ All patients demonstrated very severe chronotropic incompetence with beta-blockers; mean chronotropic index was 0.41 ± 0.14 (below 0.62 in all participants, with an average HR of 97bpm). Beta-blocker withdrawal resulted in a 30 bpm and 2.1 mL/kg/min increase in peak HR and pVO_2_, respectively. *Post hoc* analysis demonstrated that the numerical value of this benefit was the highest in participants with LV end-systolic volume below the median 14.9 mL/m^2^.^195^

The observation that administration of Ivadrabine, an I_f_ blocker that selectively lowers heart rate at rest and during exercise, diminishes pVO_2_ in HFpEF confirms the importance of equilibrium between HR and other haemodynamic and oxygen exchange parameters,^137^ though a second larger trial with ivabradine was neutral for 6MWT.^138^

## Interventions for the atrial hypertension and stressed blood volume HFpEF

### Splanchnic denervation and vasodilators

HFpEF is characterized by increased stressed blood volume.^196^ A sophisticated approach to reduce stressed blood volume and its distribution to thoracic organs is to perform splanchnic denervation to attenuate blood redistribution during splanchnic vasoconstriction with adrenergic stimulation characteristic of exercise. The REBALANCE-HF (Endovascular Ablation of the Right Greater Splanchnic Nerve in HFpEF) trial (*n* = 90 patients) showed no difference in PCWP (in one month) or exercise capacity (in 1 year), however the procedure was associated with 11% higher rate of orthostatic hypotension in instrumented vs sham-operated patients.^123^ Ongoing efforts are underway to identify suitable candidates for this intervention with careful exercise-haemodynamic phenotyping in the upright position.

Vasodilators, primarily in the form of pharmacotherapies designed to augment cGMP bioavailability, have been evaluated extensively in HFpEF. While theoretically attractive to counter ventriculo-vascular uncoupling for excess vascular stiffness and tone, cGMP augmentation strategies have not been shown to improve exercise capacity in HFpEF (*Figure 8B*).

## Interventions for the pulmonary hypertension HFpEF

### Pulmonary hypertension (PH) with HFpEF interventions

Pulmonary arterial hypertension and combined PH-HFpEF are beyond the scope of this manuscript. However, a promising approach to alleviate EI and PH-HFpEF with Levosimendan, a calcium sensitizer and K^+^-ATP channel activator,^160^ with an oral formulation currently being evaluated (NCT05983250) with 6MWD as the primary endpoint. Pulmonary artery denervation has also shown promise for lowering PVR, as has the activin inhibitor sotatercept, though careful physiologic phenotyping will be required to appropriately identify patients for therapies targeting RV-pulmonary vascular function and overlapping conditions.

### Beyond HF-directed therapies

Successful treatment of EI in HFpEF often requires a broad and holistic approach. It is important to recognize and address concomitant peripheral arterial disease, neuropathy, lymphedema, arthritis, and mental barriers (*Table 2*). Furthermore, medications that contribute to fluid accumulation and decreased mobility (pregabalin, anticholinergics, amlodipine, and non-steroidal anti-inflammatory drugs) require careful weighing of risks and benefits.

**Table 2:** Checklist after HFpEF management

Recommendations for further diagnostic examinations	
**Exclude coronary artery disease**	**Regional wall motion abnormalities** **Repolarization abnormalities** **Presence of risk factors, or suspected dyspnoea as angina equivalent** **Low stroke volume reserve with O2 pulse plateau**
**Cardiac magnetic resonance**	**Suspected infiltrative cardiomyopathy, inflammatory cardiomyopathy, iron overload or non-compaction**
**Pyp Scan** **+ serum light chains** **+ serum/urine immune fixation**	**(Bilateral) carpal tunnel syndrome, spinal stenosis, biceps tendon rupture, polyneuropathy, especially in older, lean men** **Biventricular hypertrophy, especially when pericardial effusion and/or apical sparing pattern of global longitudinal strain are present** **Low QRS voltage over left ventricular mass ratio**
**Ventilation-perfusion** **nuclear scan**	**(Exercise) pulmonary hypertension with disproportionate ventilation-perfusion mismatch (VE/VCO_2_ slope >45 or SpO_2_ < 92%)**
**High-resolution chest** **computed tomography**	**Restrictive lung function with impaired oxygen diffusion capacity with desaturation >5% during exercise**
**Pulmonary mechanical limitation**	Please see definition in Supplementary data online, *Table S1*. This finding should prompt pulmonary consultation

SpO_2_, peripheral capillary oxygen saturation; SV, stroke volume; Pyp scan, Technetium pyrophosphate scintigraphy; VE, peak ventilation; VCO_2_, CO_2_ production; VO_2_, oxygen consumption.

### Interventional trial synthesis and conclusions

In summary, clinical trials to date targeting EI in HFpEF are notable for generally modest, if any, improvement in exercise capacity with therapies that lower filling pressures. In this regard, the emerging pillars of HFpEF therapy that lower hospitalizations and attenuate adverse cardiac remodelling and excess wall stress have modest effects on exercise capacity, analogous to findings in HFrEF.^197^ These findings highlight the need for clinicians to incorporate but also look beyond these therapies, when seeking to improve EI in HFpEF (*Figure 8D*).^197^

The modest effects of HFpEF interventions directed at central haemodynamic abnormalities stand in stark contrast to cardio-centric interventions for obstructive hypertrophic cardiomyopathy (oHCM) (*Figure 8B* and *C*). It is notable how HCM—a predominantly cardiocentric disorder—demonstrates large-scale improvements in exercise capacity with therapies that directly modify cardiac structure or function (*Figure 8C*). These findings are consistent with observations that HCM is associated with a shallow CO-VO_2_ slope, reflecting predominant limitations to VO_2_ augmentation imposed by reduced CO,^198^ in contrast to HFpEF, which encompasses a heterogeneous spectrum of exercise limitation mechanisms, in which peripheral limitations are commonly observed with elevated CO-VO_2_ slopes.^199^

Notably, in a recent study of cardiac myosin inhibitor treatment of oHCM, improvements in pVO_2_ were proportionate to reductions in NTproBNP and left ventricular outflow tract gradients, further highlighting improvements in EI achievable with cardiospecific interventions in oHCM.^200^ By contrast, in HFpEF, O_2_ pathway modulation modelling studies have indicated only partial improvement in pVO_2_ for a given improvement in component variables of the O_2_ pathway.^6^ These findings should not diminish efforts to correct EI with cardio-specific interventions, but rather to do so with exercise-based phenotyping to augment potential effect sizes. For example, in PRESERVE-HR, targeting patients with severe chronotropic incompetence with beta-blocker withdrawal resulted in a 31% increase in peak HR and a 16.9% increase in pVO_2_,^195^ and studies are underway with device-based interventions with exercise-based phenotypic enrichment. At the same time, further studies are needed that are specifically directed at improving peripheral O_2_ utilization, including efforts to counter sarcopenia and lean muscle loss, promote adherence to multi-modal exercise interventions, and further explore iron repletion and other strategies to augment Ca-vO_2_.

## Conclusion and future directions

EI in HFpEF arises from a multifactorial constellation of central and peripheral abnormalities, often manifesting as layered deficits across multiple physiologic domains. Advances in high-resolution exercise-based phenotyping are aiding in dissecting the relative predominance of domain-specific contributions to EI. Therapeutic strategies that target only cardiac mechanisms have had limited success to date, but there is an overall trend in more positive trials of HFpEF interventions to augment EI in the last 5 years. Interventions to address EI in HFpEF should be considered alongside guideline-directed medical therapy directed at reducing mortality and hospitalizations. Future interventions guided by domain-based phenotyping to identify and target physiologic limitations in each patient hold promise but require further investigation.

## Supplementary Material

ehag175_Supplementary_Data

## Declarations

### Data Availability

No data were generated or analysed for or in support of this paper.
