# Supplementary material for: Heart failure and preserved ejection fraction: pathophysiology, clinical assessment, and management of exercise intolerance
Source: Eur Heart J. 2026 Apr 15;47(24):3065–101. doi: 10.1093/eurheartj/ehag175 (PMC13286652; doi:10.1093/eurheartj/ehag175)
Supplement: ehag175_Supplementary_Data [file ehag175_supplementary_data.docx]

**Table S1. Exercise Testing Parameters for Evaluation of Exercise Intolerance in HFpEF by Domain and Methodology.**

|  |  |  |  | **Exercise**  **Phase** | | | | **Domain** | | | **Modality** | | | | |  |
| --- | --- | --- | --- | --- | --- | --- | --- | --- | --- | --- | --- | --- | --- | --- | --- | --- |
| **Parameter** | **Measurement/Calculation** | **Abnormal Threshold** | **Comments and physiologic/clinical relevance to HFpEF** | **Start** | **Intermediate** | **Peak** | **Post** | **O_2_ utilization** | **Pulmonary** | **Hemodynamic** | **CPET** | **Exercise Echo** | **Exercise RHC** | **CPET-echo** | **Invasive CPET** | **Reference** |
| **Oxygen Uptake Patterns Amenable to Assessment with Noninvasive CPET** | | | | | | | | | | | | | | | | |
| Peak VO_2_ | Peak 30-second median VO_2_ during the last 90 seconds of loaded exercise | <14 - 18 ml/kg/min | Gold standard indicator of overall fitness level and prognostic marker across ejection fraction strata in HF |  |  | + |  | + | + | + | + |  |  | + | + | ^6,38-40^ |
| Peak VO_2_  (% predicted) | Percentage of predicted peak VO_2_ based on age, sex, height, and weight (i.e. Wasserman-Hansen formula) | <80% predicted | Provides useful context for VO_2_ interpretation, particularly for personalized risk assessment at extreme of age and body mass index |  |  | + |  | + | + | + | + |  |  | + | + | ^6,38-40^ |
| Aerobic Efficiency (mL/min/Watts) | ΔVO_2_/ΔWR (measurement is calculated 1 minute after incremental ramp exercise initiation) | <10 mL/min/Watt | A slope < 10±1.5 mL/min/Watt reflects increased reliance on anaerobic metabolism during incremental exercise, commonly seen in HF | + | + | + |  | + |  | + | + |  |  | + | + | ^41^ |
| VO_2_-derived Internal work (Watts) | (Unloaded exercise VO_2_ - rest VO_2_)/aerobic efficiency | >25 Watts | Metabolic cost of initiating exercise, which is elevated in individuals with obese HFpEF and limits the ability to perform external work | + |  |  |  | + |  | + | + |  |  | + | + | ^42^ |
| VO_2_ at anaerobic threshold (mL/kg/min) | VO_2_ value at VAT, identified by the inflection point in the VO_2_ and VCO_2_ slopes (V-slope method) | <9 mL/kg/min,  <40% of predicted peak VO_2_ | Reduced VO_2_ at the ventilatory anaerobic threshold is indicative of impaired submaximal exercise capacity in HFpEF |  | + |  |  | + |  | + | + |  |  | + | + | ^43^ |
| O_2_ uptake efficiency slope (L/min) | Slope of VO_2_ plotted against the logarithm of V_E_ during incremental exercise | <1.47 L/min | Indicates efficiency of VO_2_ relative to ventilation, and is relatively effort-independent | + | + | + |  | + | + | + | + |  |  | + | + | ^44^ |
| O_2_ pulse (mL/kg) | VO_2_/heart rate | <85% of age-predicted value | Reflects the product of stroke volume and peripheral O_2_ extraction that is often reduced in HFpEF, but does not further parse SV and CavO_2_ deficits | + | + | + |  | + |  | + | + |  |  | + | + | ^45,46^ |
| VO_2_ Recovery Delay (seconds) | Time elapsed from the end of exercise until VO_2_ consistently drops below peak VO_2_ | >25 sec | Longer time for VO_2_ to initially recover reflects degree of impairment in CO during exercise |  |  |  | + | + |  | + | + |  |  | + | + | ^47^ |
| VO_2_ Recovery T12.5% | Time elapsed from the end of exercise until VO_2_ is 12.5% reduced relative to baseline peak VO_2_ | ≥35 sec | Longer delay in early VO_2_ recovery reflects more impaired exercise hemodynamic, but not peripheral response, and predicts adverse outcomes independent of other CPET variables in HFpEF |  |  |  | + | + |  | + | + |  |  | + | + | ^48^ |
| **Additional Gas Exchange Variables Beyond Oxygen Uptake Measures** | | | | | | | | | | | | | | | | |
| V_E_/VCO₂ slope | Slope of the V_E_ and VCO_2_ relationship | >36 - 45 | Ventilatory inefficiency reflects V/Q mismatch, high values may signal reduced perfusion, pulmonary vascular dysfunction, or hyperventilation. Elevated values predict poor prognosis in HFpEF | + | + | + |  |  | + | + | + |  |  | + | + | ^40,49,50^ |
| O_2_ Desaturation | SpO_2_ or SaO_2_ | <88-92% | Desaturation during upright exercise in HFpEF is associated with high transpulmonary gradient and PVR, not isolated elevation in left-sided filling pressures. If mPAP/CO slope is < 3, then consider pulmonary parenchymal disease |  | + | + |  |  | + | + | + |  |  | + | + | ^51,52^ |
| Exercise Oscillatory Ventilation | Consecutive cyclic fluctuations in V_E_ with a cycle length of ~1 minute, amplitude >15% of resting V_E_, and duration> 60% of exercise duration | Categorical (yes/no) | Cyclic oscillatory breathing during exercise reflects reduced cardiac index and elevated cardiac filling pressures seen in advanced HFrEF primarily, but rarely in HFpEF |  | + | + |  |  |  | + | + |  |  | + | + | ^53,54^ |
| P_ET_CO_2_ (mmHg) | End-tidal CO_2_ in the resting state | <36 mmHg at rest  < 6-8 mmHg during exercise up to anaerobic threshold | Indicates ventilation/perfusion matching. It can be confounded by hyperventilation: rest RER >1.10 or rest RER >1.05 & P_ET_CO_2_ <28 mmHg | + | + | + |  |  | + | + | + |  |  | + | + | ^55,56^ |
| **Integration of Pulmonary Function Test Spirometry with Gas Exchange Measures** | | | | | | | | | | | | | | | | |
| Breathing reserve | 1 – (V_E_/MVV); with MVV = 40 x FEV_1_ | < 0.2 | Indicates mechanical ventilatory limitation, particularly relevant in patients with disproportionate dyspnea. Directs workup toward pulmonary referral for DLCO, bronchodilator response, and imaging |  |  | + |  |  | + |  | + |  |  | + | + | ^40,57^ |
| Dynamic Hyperinflation | Maximal inspiratory maneuver at rest, intermediate, and peak exercise | TV/IC >0.9 |  | + | + | + |  |  | + |  | + |  |  | + | + | ^57^ |
| **Exercise Echocardiography Measurements** | | | | | | | | | | | | | | | | |
| Impaired diastolic reserve | Exercise E/e′; PW Tissue Doppler | Average exE/e' >15; septal > 13 | E/e′ is specific but not sensitive for elevated filling pressures. Discordance with mPAP/CO slope may reflect differing diagnostic thresholds: the slope is more sensitive but less specific, while diastolic stress testing offers higher specificity. Combining both may improve diagnostic confidence. E and A fusion starts when the heart rate exceeds 90 bpm during exercise |  | + | ± |  |  |  | + |  | + |  | + |  | ^58-60^ |
| Exercise longitudinal systolic reserve | ExS' (septal); PW Tissue Doppler | < 10 cm/s | Contributes to CO and atrial reservoir function |  |  | + |  |  |  | + |  | + |  | + |  | ^61-63^ |
| Impaired chronotropic reserve | (peak HR - rest HR)/([220-age] – rest HR) | <0.8 or <0.62 (on BB) | Chronotropic incompetence or inability to access higher heart rates during exercise | + |  | + |  |  |  | + | + | + | + | + | + | ^64^ |
| Myocardial Ischemia | Regional wall motion abnormalities/ ST segment changes / VO_2_ plateau dynamics | Present | Consider ischemic work-up if plateau in VO_2_ or a fall in O_2_ pulse is observed, although these findings are nonspecific due to central and peripheral contributions. A true SV decrease (if available) is more specific. Execho has high specificity but limited sensitivity. Coronary CT may be appropriate in HFpEF patients with refractory symptoms, especially when risk factors are present and there is no known ASCVD —in situations where standard GDMT is not yet initiated |  |  | + | + |  |  | + | ± | + |  | + |  | ^40^ |
| LVOT obstruction | LVOT gradient by CW Doppler | >30 mmHg | Should prompt diagnosis of oHCM and targeted treatments for oHCM |  |  | + | + |  |  | + |  | + |  | + |  | ^58^ |
| Dynamic mitral regurgitation | MR grade increase | Increase ≥1 grade; Δ ERO +5-13 mm² | Prognostic value; limited data on AFMR as therapeutic target; might have independent prognostic value |  | + | ± |  |  |  | + |  | + |  | + |  | ^58,65,66^ |
| Increased lung water | B-lines by lung ultrasound; becomes positive with ≥3 B lines per zone makes a positive zone | ≥1 zone with ≥3 B-lines (one positive zone) on each hemithorax not present during exercise | Pulmonary congestion observed commonly in HFpEF, particularly during supine exercise |  |  | + | + |  |  | + |  | + |  | + |  | ^51,67^ |
| **Invasive Exercise Hemodynamic Measurements** | | | | | | | | | | | | | | | | |
| Exercise cardiac output Reserve | (140 × CO peak) / predicted VO_2_ peak [mL/min] CO peak / [(CO rest + (VO_2__rest – VO_2_ peak) × 6)] (values in L/min unless otherwise noted) | < 0.8 | The method based on a normal CO/VO_2_ slope of 6 may be more appropriate for submaximal test (RER < 1.05) |  |  | + |  |  |  | + |  | + |  | + | + | ^39,40,57,68^ |
| Exercise stroke volume | Stroke volume calculated as CO obtained by direct Fick CO / HR or from LVOT area × VTI by pulsed-wave Doppler (exEcho) | Peak SV <42 mL/m² ΔSV <20% | Reflects left ventricular contractile reserve function, but is dependent on pre- and afterload |  |  | + |  |  |  | + |  | + |  | + | + | ^58,69,70^ |
| Cardiac Power Output | (mean arterial pressure - RAP) × CO × 0.00222; RAP can be estimated or neglected when evaluated non-invasively | <2.0 Watt | Prognostic value; correction of CO for afterload; Reflects left ventricular contractile reserve function |  |  | + |  |  |  | + |  | + |  | + | + | ^71,72^ |
| Pulmonary hypertension, absolute pressures | absolute value of mPAP; mPAP can be estimated from TRG (+ 10 mmHg or estimated RAP) by the Chemla Formula (0,61*estimated sPAP + 2) | TRV >3.4 m/s or mPAP 30 mmHg at 25 Watt | When both the PAP/CO slope and absolute pressures are elevated, this indicates substantial hemodynamic burden |  |  | + |  |  |  | + |  | + | + | + | + | ^60,73^ |
| Pulmonary hypertension, corrected for slope | Slope of PAP vs. CO calculated from ≥ 2 data pairs from rest to peak exercise, either invasive or noninvasive; non-invasive slope: CO by LVOT Doppler and diameter * HR and mPAP by the Chemla formula. Adding a RAP estimate does not improve correlation with outcome of invasive PAP/CO slope | >3 mmHg/L/min  diagnostic  >3.5 mmHg/L/min optimal prognostic cutoff for non-invasive sloe | The PAP/CO slope reflects pulmonary vascular–LA compliance and is prognostic beyond absolute pressures, which are more effort-dependent. A high slope with low pressures may indicate high TPR, but hemodynamics may not limit exercise (e.g. HFpEF + COPD). Single-point ratios with exEcho might be more reproducible and just as prognostically informative | + | + | + |  |  |  | + |  | + |  | + | + | ^50,72,74,75^ |
| PCWP/CO slope (mmHg/L/min) | Slope of PCWP vs. CO calculated from minute-by-minute values from rest to peak exercise | >2 mmHg/L/min | Integrated measure of left heart filling pressure relative to cardiac performance. Differentiates HFpEF from control and predicts CV outcomes independently from resting HD values | + | + | + |  |  |  | + |  |  |  |  | + | ^11^ |
| PCWP/Workload/Kg  (PCWL) (mmHg/W/kg) | Exercise peak PCWP indexed to workload and body weight (PCWL= peak exercise PCWP/workload/ body weight) | >25.5 mmHg/W/kg | Higher risk of all-cause mortality when high PCWL, regardless of resting PCWP being normal or elevated |  |  | + |  |  |  | + |  |  | + |  | + | ^76^ |
| PCWP rest / PCWP exercise | Rest and Exercise PCWP | >15 mmHg (rest); >25 mmHg (exercise) | The earlier (lower workload, e.g., 25 watts) the threshold is crossed, the more specific the finding. Associated with worse outcomes when measured in the supine position | + | + | + |  |  |  | + |  |  | + |  | + | ^35^ |
| CO/VO_2_ slope | Cardiac output per VO₂ increment | >5 | In patients with normal CO reserve, a higher threshold for CO/VO_2_ slope > 7 can indicate low O_2_ utilization and predominantly peripheral limitation to exercise performance. | + | + | + |  | + |  | + |  |  |  | + | + | ^70,77-81^ |
| a–vO_2_Diff /Hb | Direct measurement: a–vO_2_Diff with O_2_ content= 1.34 × Hb × SO_2_ + 0.003 × PO_2_  or estimated from Fick:  (100 × VO_2_) / (CO × Hb) | <0.8 | E.g., a–vO_2_Diff of 10 mL/dL is normal when Hb is 10 g/dL. Non-invasive a–vO_2_Diff by VO_2_ from CPET, CO from exEcho, 100 for unit conversion | + |  | + |  | + |  |  |  |  |  | + | + | ^82,83^ |
| Abbreviations: a–vO_2_Diff, arteriovenous oxygen difference; AFMR, atrial functional mitral regurgitation; ASCVD, atherosclerotic cardiovascular disease; BB, beta-blocker; BMI, body mass index; CO, cardiac output; CPET, cardiopulmonary exercise testing; CPETecho, combined cardiopulmonary exercise testing and echocardiography; CW Doppler, continuous wave Doppler; DLCO, diffusing capacity of the lung for carbon monoxide; ERO, effective regurgitant orifice; ExEcho, exercise echocardiography; ExS′, exercise-induced septal systolic velocity; FEV_1_, forced expiratory volume in one second; GDMT, guideline-directed medical therapy; Hb, hemoglobin; HF, heart failure; HFpEF, heart failure with preserved ejection fraction; HR, heart rate; IC, inspiratory capacity; LVOT, left ventricular outflow tract; mPAP, mean pulmonary artery pressure; MR, mitral regurgitation; MVV, maximal voluntary ventilation; O_2_, oxygen; PCWP, pulmonary capillary wedge pressure; PCWL, pulmonary capillary wedge pressure indexed to workload and body weight; PETCO_2_, partial pressure of end-tidal carbon dioxide; PO_2_, partial pressure of oxygen; PW, pulsed-wave; RAP, right atrial pressure; RER, respiratory exchange ratio; sPAP, systolic pulmonary artery pressure; SpO_2_, peripheral capillary oxygen saturation; SV, stroke volume; TRG, tricuspid regurgitation gradient; TRV, tricuspid regurgitation velocity; TV, tidal volume; VAT, ventilatory anaerobic threshold; VE, minute ventilation; VE/VCO_2_, minute ventilation to carbon dioxide production; VO_2_, oxygen uptake; VCO_2_, carbon dioxide output; V/Q, ventilation/perfusion; VTI, velocity-time integral. | | | | | | | | | | | | | | | | |

**References:**

39. Wasserman K, Hansen J, Sue DY, Stringer W, Sietsema K, Sun X-G, et al. Principles of exercise testing and interpretation: Including pathophysiology and clinical applications: Fifth edition2011. 1-592 p.

40. Guazzi M, Adams V, Conraads V, Halle M, Mezzani A, Vanhees L, et al. EACPR/AHA Scientific Statement. Clinical recommendations for cardiopulmonary exercise testing data assessment in specific patient populations. Circulation. 2012;126(18):2261-74.

41. Tanabe Y, Nakagawa I, Ito E, Suzuki K. Hemodynamic basis of the reduced oxygen uptake relative to work rate during incremental exercise in patients with chronic heart failure. Int J Cardiol. 2002;83(1):57-62.

42. Shah RV, Schoenike MW, Armengol de la Hoz MA, Cunningham TF, Blodgett JB, Tanguay M, et al. Metabolic Cost of Exercise Initiation in Patients With Heart Failure With Preserved Ejection Fraction vs Community-Dwelling Adults. JAMA Cardiol. 2021;6(6):653-60.

43. Beaver WL, Wasserman K, Whipp BJ. A new method for detecting anaerobic threshold by gas exchange. J Appl Physiol (1985). 1986;60(6):2020-7.

44. Hollenberg M, Tager IB. Oxygen uptake efficiency slope: an index of exercise performance and cardiopulmonary reserve requiring only submaximal exercise. J Am Coll Cardiol. 2000;36(1):194-201.

45. Oliveira RB, Myers J, Araujo CG, Arena R, Mandic S, Bensimhon D, et al. Does peak oxygen pulse complement peak oxygen uptake in risk stratifying patients with heart failure? Am J Cardiol. 2009;104(4):554-8.

46. Milani M, Bekhuis Y, Falter M, Moura-Ferreira S, Hoedemakers S, L Hoyes W, et al. Does oxygen pulse represent a reliable surrogate for stroke volume during exercise? Insights from cardiopulmonary exercise test combined with echocardiography or invasive measurements. European Journal of Preventive Cardiology. 2025;32(Supplement_1).

47. Bailey CS, Wooster LT, Buswell M, Patel S, Pappagianopoulos PP, Bakken K, et al. Post-Exercise Oxygen Uptake Recovery Delay: A Novel Index of Impaired Cardiac Reserve Capacity in Heart Failure. JACC Heart Fail. 2018;6(4):329-39.

48. Campain J, Griskowitz C, Newlands C, Claggett BL, Kulac IJ, McGinnis S, et al. Characterization and Application of Novel Exercise Recovery Patterns That Reflect Cardiac Performance: A Substudy of the SEQUOIA-HCM Trial. Circulation. 2025.

50. Caravita S, Faini A, Deboeck G, Bondue A, Naeije R, Parati G, et al. Pulmonary hypertension and ventilation during exercise: Role of the pre-capillary component. J Heart Lung Transplant. 2017;36(7):754-62.

54. Murphy RM, Shah RV, Malhotra R, Pappagianopoulos PP, Hough SS, Systrom DM, et al. Exercise oscillatory ventilation in systolic heart failure: an indicator of impaired hemodynamic response to exercise. Circulation. 2011;124(13):1442-51.

55. Myers J, Gujja P, Neelagaru S, Hsu L, Vittorio T, Jackson-Nelson T, et al. End-tidal CO2 pressure and cardiac performance during exercise in heart failure. Med Sci Sports Exerc. 2009;41(1):19-25.

56. Nayor M, Xanthakis V, Tanguay M, Blodgett JB, Shah RV, Schoenike M, et al. Clinical and Hemodynamic Associations and Prognostic Implications of Ventilatory Efficiency in Patients With Preserved Left Ventricular Systolic Function. Circ Heart Fail. 2020;13(5):e006729.

58. Lancellotti P, Pellikka PA, Budts W, Chaudhry FA, Donal E, Dulgheru R, et al. The clinical use of stress echocardiography in non-ischaemic heart disease: recommendations from the European Association of Cardiovascular Imaging and the American Society of Echocardiography. Eur Heart J Cardiovasc Imaging. 2016;17(11):1191-229.

59. Obokata M, Kane GC, Reddy YN, Olson TP, Melenovsky V, Borlaug BA. Role of Diastolic Stress Testing in the Evaluation for Heart Failure With Preserved Ejection Fraction: A Simultaneous Invasive-Echocardiographic Study. Circulation. 2017;135(9):825-38.

60. Pieske B, Tschope C, de Boer RA, Fraser AG, Anker SD, Donal E, et al. How to diagnose heart failure with preserved ejection fraction: the HFA-PEFF diagnostic algorithm: a consensus recommendation from the Heart Failure Association (HFA) of the European Society of Cardiology (ESC). Eur Heart J. 2019;40(40):3297-317.

62. Verwerft J, Verbrugge FH, Claessen G, Herbots L, Dendale P, Gevaert AB. Exercise Systolic Reserve and Exercise Pulmonary Hypertension Improve Diagnosis of Heart Failure With Preserved Ejection Fraction. Front Cardiovasc Med. 2022;9:814601.

63. Claeys M, Petit T, La Gerche A, Herbots L, Claus P, De Bosscher R, et al. Impaired biventricular contractile reserve in patients with diastolic dysfunction: insights from exercise stress echocardiography. Eur Heart J Cardiovasc Imaging. 2022;23(8):1042-52.

64. Brubaker PH, Kitzman DW. Chronotropic incompetence: causes, consequences, and management. Circulation. 2011;123(9):1010-20.

65. Falter M, Bekhuis Y, Hoedemakers S, Moura-Ferreira S, Coosemans S, L'hoyes W, et al. An elevated mPAP over CO slope by exercise echocardiography predicts outcome in unexplained dyspnea. European Heart Journal. 2024;45(Supplement_1).

66. Dhont S, Ferreira SM, Galloo X, Martens P, Meekers E, Tartaglia K, et al. Angiotensin Receptor Neprilysin Inhibitor in Heart Failure with Preserved Ejection Fraction and Secondary Mitral Regurgitation: Design and Rationale of the PRAISE-MR trial. Journal of Cardiac Failure. 2025.

67. Reddy YNV, Obokata M, Wiley B, Koepp KE, Jorgenson CC, Egbe A, et al. The haemodynamic basis of lung congestion during exercise in heart failure with preserved ejection fraction. Eur Heart J. 2019;40(45):3721-30.

68. Jain CC, Borlaug BA. Performance and Interpretation of Invasive Hemodynamic Exercise Testing. Chest. 2020;158(5):2119-29.

69. Higginbotham MB, Morris KG, Williams RS, McHale PA, Coleman RE, Cobb FR. Regulation of stroke volume during submaximal and maximal upright exercise in normal man. Circ Res. 1986;58(2):281-91.

70. Martens P, Herbots L, Timmermans P, Verbrugge FH, Dendale P, Borlaug BA, et al. Cardiopulmonary Exercise Testing with Echocardiography to Identify Mechanisms of Unexplained Dyspnea. J Cardiovasc Transl Res. 2022;15(1):116-30.

71. Takizawa D, Harada T, Obokata M, Kagami K, Sorimachi H, Yuasa N, et al. Pathophysiologic and prognostic importance of cardiac power output reserve in heart failure with preserved ejection fraction. European Heart Journal - Cardiovascular Imaging. 2023;25(2):220-8.

72. Falter M, Bekhuis Y, L'Hoyes W, Milani M, Hoedemakers S, Soens L, et al. Exercise Echocardiography for Risk Stratification in Unexplained Dyspnea: the Incremental Value of the mPAP/CO slope. J Am Soc Echocardiogr. 2025.

74. Kovacs G, Herve P, Barbera JA, Chaouat A, Chemla D, Condliffe R, et al. An official European Respiratory Society statement: pulmonary haemodynamics during exercise. Eur Respir J. 2017;50(5).

75. Bekhuis YG, Falter M, Milani M, Hoedemakers S, Moura-Ferreira S, Verbrugge F, et al. Mean pulmonary arterial pressure over cardiac output slope for risk stratification in patients with unexplained dyspnea: does the math matter? European Heart Journal - Cardiovascular Imaging. 2025;26(Supplement_1).

76. Dorfs S, Zeh W, Hochholzer W, Jander N, Kienzle RP, Pieske B, et al. Pulmonary capillary wedge pressure during exercise and long-term mortality in patients with suspected heart failure with preserved ejection fraction. Eur Heart J. 2014;35(44):3103-12.

78. Skow RJ, Sarma S, MacNamara JP, Bartlett MF, Wakeham DJ, Martin ZT, et al. Identifying the Mechanisms of a Peripherally Limited Exercise Phenotype in Patients With Heart Failure With Preserved Ejection Fraction. Circulation: Heart Failure.0(0):e011693.

80. Pugliese NR, Mazzola M, Fabiani I, Gargani L, De Biase N, Pedrinelli R, et al. Haemodynamic and metabolic phenotyping of hypertensive patients with and without heart failure by combining cardiopulmonary and echocardiographic stress test. Eur J Heart Fail. 2020;22(3):458-68.

82. Del Punta L, De Biase N, Armenia S, Di Fiore V, Maremmani D, Gargani L, et al. Combining cardiopulmonary exercise testing with echocardiography: a multiparametric approach to the cardiovascular and cardiopulmonary systems. European Heart Journal - Imaging Methods and Practice. 2023;1(1).

83. Verwerft J, Bertrand PB, Claessen G, Herbots L, Verbrugge FH. Cardiopulmonary Exercise Testing With Simultaneous Echocardiography: Blueprints of a Dyspnea Clinic for Suspected HFpEF. JACC Heart Fail. 2023;11(2):243-9.

84. Tan YT, Wenzelburger F, Lee E, Heatlie G, Leyva F, Patel K, et al. The pathophysiology of heart failure with normal ejection fraction: exercise echocardiography reveals complex abnormalities of both systolic and diastolic ventricular function involving torsion, untwist, and longitudinal motion. J Am Coll Cardiol. 2009;54(1):36-46.
